# Siglec-15 recognition of sialoglycans on tumor cell lines can occur independently of sialyl Tn antigen expression

**DOI:** 10.1093/glycob/cwaa048

**Published:** 2020-05-30

**Authors:** Gavuthami Murugesan, Viviana G Correia, Angelina S Palma, Wengang Chai, Chunxia Li, Ten Feizi, Eva Martin, Brigitte Laux, Alexandra Franz, Klaus Fuchs, Bernd Weigle, Paul R Crocker

**Affiliations:** 1 Division of Cell Signalling and Immunology, School of Life Sciences, University of Dundee, Dundee, DD1 5EH, United Kingdom; 2 Applied Molecular Biosciences Unit, Faculty of Science and Technology, NOVA University of Lisbon, Lisbon, Portugal; 3 Applied Molecular Biosciences Unit, Department of Chemistry, Faculty of Science and Technology, NOVA University of Lisbon, Lisbon, Portugal; 4 Glycosciences Laboratory, Imperial College London, London, United Kingdom; 5 Key Laboratory of Marine Drugs, Ministry of Education, School of Medicine and Pharmacy and Shandong Provincial Key laboratory of Glycoscience and Glycoengineering, Ocean University of China, Qingdao 266003, China; 6 Drug Discovery Sciences, Boehringer Ingelheim Pharma GmbH & Co KG, Birkendorfer Str. 65, 88397 Biberach/Riss, Germany; 7 Cancer Immunology & Immune Modulation, Boehringer Ingelheim Pharma GmbH & Co KG, Birkendorfer Str. 65, 88397 Biberach/Riss, Germany; 8 Biotherapeutics Discovery, Boehringer Ingelheim Pharma GmbH & Co KG, Birkendorfer Str. 65, 88397 Biberach/Riss, Germany

**Keywords:** cancer, sialic acid, siglec

## Abstract

Siglec-15 is a conserved sialic acid-binding Ig-like lectin expressed on osteoclast progenitors, which plays an important role in osteoclast development and function. It is also expressed by tumor-associated macrophages and by some tumors, where it is thought to contribute to the immunosuppressive microenvironment. It was shown previously that engagement of macrophage-expressed Siglec-15 with tumor cells expressing its ligand, sialyl Tn (sTn), triggered production of TGF-β. In the present study, we have further investigated the interaction between Siglec-15 and sTn on tumor cells and its functional consequences. Based on binding assays with lung and breast cancer cell lines and glycan-modified cells, we failed to see evidence for recognition of sTn by Siglec-15. However, using a microarray of diverse, structurally defined glycans, we show that Siglec-15 binds with higher avidity to sialylated glycans other than sTn or related antigen sequences. In addition, we were unable to demonstrate enhanced TGF-β secretion following co-culture of Siglec-15-expressing monocytic cell lines with tumor cells expressing sTn or following Siglec-15 cross-linking with monoclonal antibodies. However, we did observe activation of the SYK/MAPK signaling pathway following antibody cross-linking of Siglec-15 that may modulate the functional activity of macrophages.

## Introduction

Siglecs are sialic acid binding Ig-like lectins expressed mainly on the surface of hemopoietic and immune cells (Crocker et al. 2007; Crocker and Redelinghuys 2008). Siglec-15 is highly conserved across vertebrate evolution (Angata et al. 2007; Angata 2020) and consists of an N-terminal sialic acid-binding V-set domain, a C2-set domain, a positively charged transmembrane region and a cytosolic tail. Through its charged transmembrane region, Siglec-15 becomes associated with the immunoreceptor tyrosine-based activation motif (ITAM) adaptor proteins, DAP10 or DAP12, and has been shown to trigger cell signaling following cross-linking at the cell surface (Angata et al. 2007; Takamiya et al. 2013; Stuible et al. 2014). Under steady-state conditions, it is primarily expressed on osteoclasts following stimulation of progenitors with RANKL and CSF-1 (Hiruma et al. 2011; Ishida-Kitagawa et al. 2012; Stuible et al. 2014). Consistent with a functional role of Siglec-15 in bone resorption, Siglec-15-deficient mice exhibit a mild osteopetrotic phenotype (Hiruma et al. 2013; Kameda et al. 2013) and monoclonal antibodies to Siglec-15 can be used to treat osteoporosis (Sato et al. 2018). Besides osteoclasts, Siglec-15 is expressed on a subset of tissue macrophages (Angata et al. 2007) and is upregulated on tumor-associated macrophages (TAMs) (Takamiya et al. 2013; Wang et al. 2019). In addition to TAMs, some tumor cells upregulate Siglec-15 expression where it contributes to the immunosuppressive microenvironment by blocking CD8 T cell proliferation (Wang et al. 2019). Recent genome-wide association studies have shown that Siglec-15 can function as a susceptibility factor to infectious diseases, including recurrent vulvovaginal candidiasis (Jaeger et al. 2019) and pulmonary tuberculosis (Bhattacharyya et al. 2019).

The initial studies with recombinant Siglec-15 suggested that the sialyl Tn (sTn) antigen (Neu5Acα2,6GalNAc) is a preferred glycan ligand for mouse and human Siglec-15 (Angata et al. 2007). STn is a truncated O-glycan that is overexpressed on mucins in carcinomas such as breast, ovarian, lung and gastric cancers (Julien et al. 2012; Munkley 2016). ST6GalNAc-I is a sTn synthase that catalyzes α2,6-sialyl linkage to GalNAcα-O-Ser/Thr (Marcos et al. 2004; Julien et al. 2012). In normal cells, T synthase competes with ST6GalNAc-I by adding Gal to GalNAc giving rise to core 1 or T antigen, and this activity of T synthase is dependent on a chaperone known as Cosmc. In cancers, mutation of Cosmc results in elevated sTn levels that are associated with poor prognosis and outcome (Beatson et al. 2015; Munkley 2016). Interestingly, when lung carcinoma cells overexpressing the sTn synthase were co-cultured with THP-1 macrophage-like cells overexpressing Siglec-15, this led to increased production of immunosuppressive TGF-β by the THP-1 cells (Takamiya et al. 2013). Taken together, these observations suggest that interactions between sTn and Siglec-15 can play important roles in crosstalk between tumor cells and the immune system.

In the present study, we have further investigated the interaction between Siglec-15 and sTn on tumor cells and its functional consequences. Based on binding assays with lung and breast cancer cell lines and glycan-modified cells, we did not observe evidence for recognition of sTn by Siglec-15 in these cell models. However, using a structurally diverse and sequence-defined glycan microarray, we have shown that Siglec-15 exhibits higher avidity to sialylated glycans other than sTn. In addition, we were unable to demonstrate enhanced TGF-β secretion following Siglec-15 cross-linking in overexpressing THP-1 cells, but we did observe activation of the SYK/MAPK signaling pathway that may modulate their functional activity.

## Results

### Siglec-15 binds lung and breast cancer cell lines

**Fig. 1:**
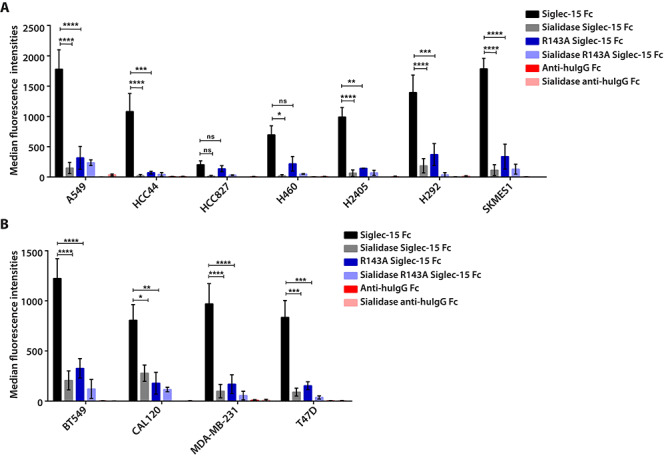
Siglec-15-Fc binds lung and breast adenocarcinoma cell lines. For the binding assay, recombinant Siglec-15 human IgG1 Fc fusion protein (Siglec-15-Fc) wildtype (1 μg/mL) or R143A mutant (1 μg/mL) was mixed with goat anti-human IgG Fc-FITC (1 μg/mL) to prepare immune-complexes. Lung and breast carcinoma cell lines, pretreated with or without sialidase, were incubated with Siglec-15-Fc immune complexes. Negative controls were incubations with anti-human IgG Fc-FITC without the addition of Siglec-15-Fc. The binding of Siglec-15-Fc to cancer cells was analyzed by flow cytometry. The results are mean ± SEM of fluorescence intensities (MFI) measured from three independent experiments for each lung **(A)** and breast **(B)** cancer cell line, relative to the nonstained control. ^*^, P < 0.05; ^**^, P < 0.01; ^***^, P < 0.005; ns, not significant.

We tested a panel of human nonsmall cell lung cancer and breast carcinoma cell lines for binding of precomplexed human Siglec-15-IgG1-Fc fusion protein. Siglec-15-Fc protein bound lung cancer cells with varying avidities (Figure 1A and Supplementary Figure S1A). All the breast carcinoma cell lines tested bound Siglec-15-Fc at a similar level (Figure 1B and Supplementary Figure S1B). In all cases, Siglec-15-Fc binding was sialic acid-dependent as pretreatment of the cells with sialidase strongly reduced binding (Figure 1). Canonical Siglec binding to glycan ligands requires a conserved Arg residue that forms a salt bridge with the carboxylate of sialic acid (Crocker et al. 2007). The binding of Siglec-15-Fc to cancer cell lines was greatly reduced on mutation of the key Arg143 to Ala (Figure 1A and B and Supplementary Figure S1C and D). Unlike a previous study (Angata et al. 2007), however, this mutation did not completely abrogate Siglec-15 binding to cancer cell lines.

### Siglec-15 binds ligands other than sialyl Tn on cancer cell lines

**Fig. 2:**
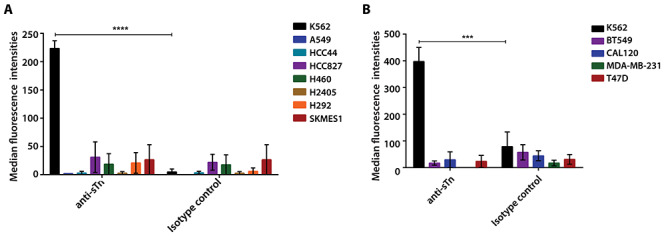
Sialyl Tn is not expressed on lung and breast cancer cell lines that bind Siglec-15-Fc. Expression of sTn on lung **(A)** and breast **(B)** cancer cell lines was detected by anti-sTn antibody (clone 3F1) (7.5 μg/mL) followed by APC conjugated anti-mouse IgG. An isotype matched mouse IgG was used as the negative control. The results are plotted as median fluorescence intensities ± SEM from 3 independent experiments, relative to the signal with secondary antibody alone. ^***^, P < 0.005; ^****^, P < 0.001; ns, not significant.

STn was reported to be an important glycan ligand for Siglec-15 in binding assays using a set of PAA-biotin glycans and cell-based studies (Angata et al. 2007, Takamiya et al. 2013). To investigate whether sTn is expressed on the lung and breast cancer cell lines that bound Siglec-15-Fc fusion protein, we performed flow cytometry with a commercial antibody (clone 3F1) against sTn. The leukemic cell line, K562, was used as a positive control, as it is known to express endogenous sTn (Sewell et al. 2006). 3F1 stained K562 cells in a concentration-dependent manner and this was strongly reduced following treatment of cells with sialidase (Supplementary Figure S2). Moreover, the binding was inhibited in the presence of sTn-PAA-biotin glycoconjugate (data not shown). In striking contrast to K562 cells, we could not detect sTn expression using 3F1 on any of the lung and breast cancer cell lines, including those that bound strongly to Siglec-15-Fc (Figure 2 and Supplementary Figure S3).

**Fig. 3:**
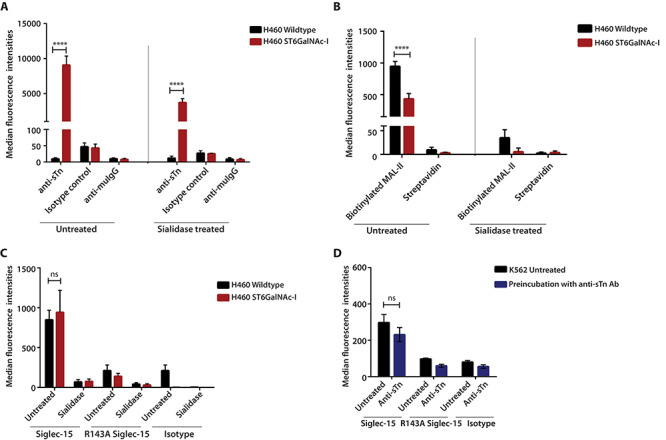
Enhanced sialyl Tn expression does not lead to increased Siglec-15-Fc binding. (**A**) NCI-H460 wildtype cells and NCI-H460 cells over-expressing ST6GalNAc-I were analyzed for sTn expression using mouse anti-sTn antibody (clone 3F1) followed by APC-conjugated anti-mouse IgG. Specificity of antibody binding was analyzed using cells pretreated with sialidase to destroy the epitope. An isotype-matched mouse IgG was used as the negative control. (**B**) NCI-H460 wildtype and ST6GalNAc-I-expressing cells were stained using biotinylated MAL-II lectin (5 μg/mL) followed by streptavidin-FITC. Staining with streptavidin-FITC alone was used as the negative control. (**C**) H460 wildtype and ST6GalNAc-I-expressing cells, untreated or pretreated with sialidase, were incubated with Siglec-15-Fc wildtype and R143A mutant precomplexed with anti-human IgG1 Fc-FITC (at ratio 1:1) and analyzed by flow cytometry. Negative controls were incubations with human IgG1 precomplexed with anti-human IgG1 Fc-FITC (at ratio 1:1). (**D**) K562 cells, preincubated with or without anti-sTn antibody (20 μg/mL), were analyzed for Siglec-15 binding as described in (C) above. Results are plotted as median fluorescence intensity (MFI) ± SEM for 3 independent experiments, relative to the signal with secondary antibody alone. ^****^, P < 0.001; ns, not significant.

To further investigate whether sTn can function as a cell surface ligand for Siglec-15, we overexpressed sTn synthase, ST6GalNAc-I, in the H460 lung cancer cell line. This cell line was selected because it bound at low to intermediate levels to Siglec-15 and should allow any increased binding to be readily evident. Overexpression of ST6GalNAc-I led to a greater than 70-fold increase in surface levels of sTn as detected by 3F1 antibody, which was greatly reduced by sialidase pretreatment of the cells (Figure 3A and Supplementary Figure S4A). High expression of sTn was also associated with altered α2,3 sialylation, as evident from the reduced MALII lectin binding (Figure 3B and Supplementary Figure S4B). However, overexpression of sTn in H460 cells had no impact on Siglec-15-Fc binding compared to the wild type cells (Figure 3C and Supplementary Figure S4C). Moreover, the binding of Siglec-15-Fc to K562 cells that express endogenous sTn was not affected by pretreatment with the 3F1 anti-sTn monoclonal antibody expected to block sTn-dependent binding (Figure 3D and Supplementary Figure S4D). Therefore, in contrast to previous studies, our results suggest that sTn does not function effectively as a cell surface ligand for Siglec-15.

### Identification of glycan ligands for Siglec-15

**Fig. 4:**
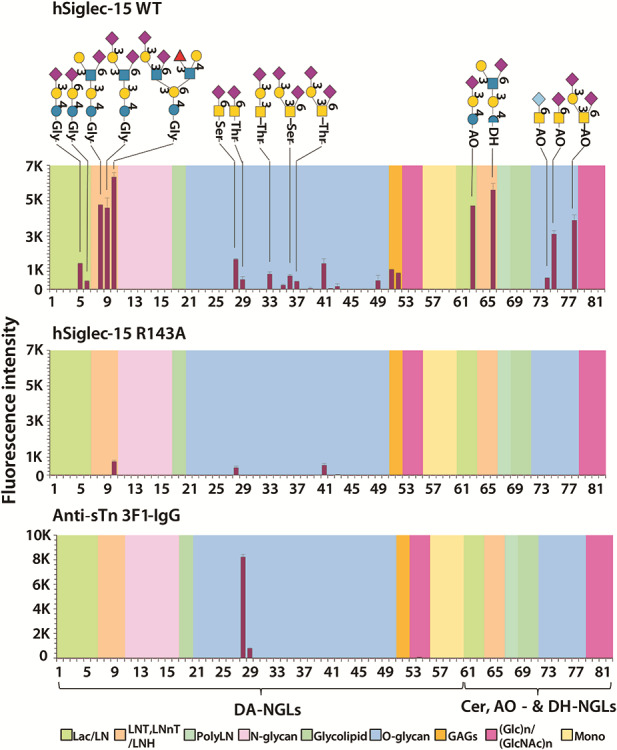
Glycan microarray analysis of human Siglec-15-Fc chimeras and anti-sTn 3F1 mAb. The Siglec-15-Fc chimeras were analyzed as precomplexes with biotinylated anti-human IgG antibody at 1:1 ratio (by weight) and at a final concentration of 2 μg/mL. The anti-sTn 3F1-IgG was analyzed at 50 μg/mL. The results are the means of fluorescence intensities of duplicate glycan probe spots, printed at 5 fmol/spot. The probes are grouped according to their backbone-type sequences: disaccharide-based, lactose (Lac) and lactosamine (LN); tetrasaccharide- or hexasaccharide-based, lacto-*N*-tetraose (LNT), lacto-*N*-*neo*-tetraose (LNnT), lacto-*N*-hexaose (LNH); poly-*N*-acetyllactosamine (PolyLN); *N*-glycans; glycolipid; *O*-glycan; glycosaminoglycans (GAGs); Glc and GlcNAc homo-oligomers; monosaccharides. The probe sequences are listed in Supplementary Table SI.

To identify glycan ligands for Siglec-15 other than sTn, we performed glycan array analyses of Fc-chimeras of human Siglec-15 wildtype and Arg143Ala mutant and compared the results with those using the sTn specific 3F1 antibody (Figure 4). We used a newly constructed glycan microarray consisting of 82 sequence-defined neoglycolipid (NGL) probes. Sixty of these were derived from synthetic amino-terminating glycans and naturally derived glyco-amino acids (Supplementary Table SI). These were conjugated to a newly synthesized aldehyde-functionalized phospholipid reagent *N*-(4-formylbenzamide)-1,2-dihexadecyl-*sn*-glycero-3-phosphoethanolamine, abbreviated to DA (W Chai and colleagues, submitted); they are designated DA-NGLs (Supplementary Table SII). As controls, 22 conventional NGLs and glycolipids were included.

Binding of Siglec-15-Fc was observed to the sTn, α2,6-sialyl GalNAc-antigen linked to Ser or Thr (DA-NGL probes 28 and 29, Figure 4 and Supplementary Table SII). There was binding also to aminooxy AO-NGLs derived from the *N*-glycolyl and *N*-acetyl forms of the reducing disaccharide obtained from bovine submaxillary mucin (Chai et al. 1992) (probes 74 and 75); and to the α2,3-sialyl and α2,3/α2,6-disialyl core 1 linked to Ser or Thr (DA-NGL probes 33, 36 and 37) and to the AO-NGL analogue (probe 78). Siglec-15-Fc binding could also be detected to α2,3- and α2,6-sialyl lactose (probes 5, 6 and 63). However, the predominant binding was detected to α2,6-sialyl glycans with lacto-*N*-tetraose and lacto-*N*-hexaose backbone sequences. These included the α2,6- monosialyl LSTb and the α2,3/α2,6-disialyl DSLNT and DSMFLNH (probes 8–10 and 66, Figure 4 and Supplementary Table SII). With the Arg143 to Ala mutant the binding was markedly reduced, but not abolished. In sum, Siglec-15 binding contrasts with the binding observed with the 3F1-IgG antibody, which is restricted to the α2,6-sialylated GalNAc, sTn antigen. These results are consistent with the cell-based studies above.

### Cross-linking Siglec-15 on monocytic cells did not enhance TGF-β secretion

**Fig. 5:**
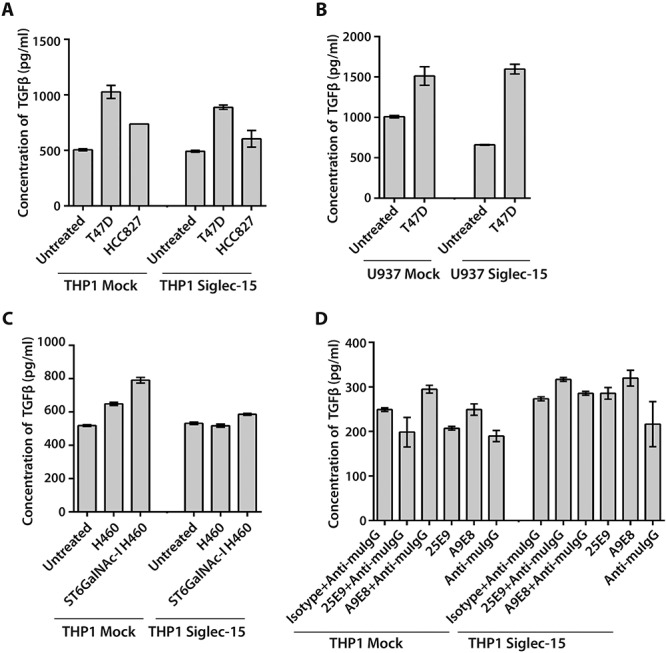
Effect of cross-linking Siglec-15 on TGF-β secretion in monocytic cells. (**A**) Lung cancer cell lines T47D and HCC827 that show differential expression of Siglec-15 ligands were co-cultured with THP-1 mock and human Siglec-15-expressing monocytic cells for 24 h in serum free medium. (**B**) T47D cells were co-cultured with U937 mock and human Siglec-15-expressing cells. (**C**) H460 wildtype and sTn-expressing (ST6GalNAc-I) cells were co-cultured with THP-1 mock and Siglec-15-expressing cells. (**D**) Mock-transfected and Siglec-15-expressing THP-1 cells were incubated with monoclonal antibodies against human Siglec-15 and crosslinked with anti-mouse IgG F(ab’)_2_ secondary antibody. The cells were cultured in macrophage serum-free medium for 24 h. To determine secretion of TGF-β, supernatants were concentrated as described in the Materials and Methods, acidified to release active TGF-β, neutralized and analyzed using Human TGF-β1 Quantikine ELISA kit according to the manufacturer’s instructions. The results are mean concentrations ± SD for two independent experiments.

Previously it was shown that co-culture of sTn-expressing lung cancer cells with either M-CSF-induced human macrophages or Siglec-15-transfected THP-1 monocyte-like cells promoted secretion of TGF-β (Takamiya et al. 2013). We performed similar experiments using THP-1 and U937 monocyte-like cells stably expressing human Siglec-15 (Supplementary Figure S5) co-cultured with lung and breast cancer cells. However, we failed to see any evidence for Siglec-15-dependent increase in TGF-β production under the conditions used (Figure 5A–C).

**Fig. 6:**
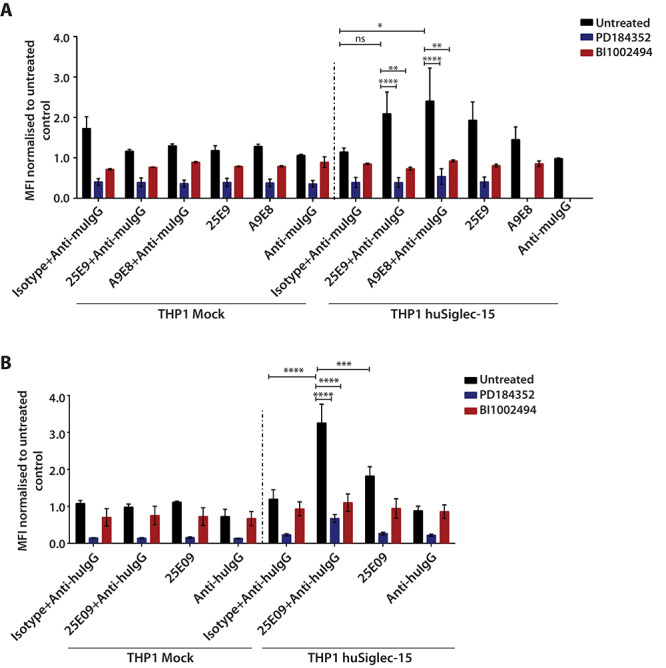
Effect of cross-linking Siglec-15 on ERK activation. (**A**) Mock-transfected THP-1 and Siglec-15-expressing cells were incubated with mouse IgG antibodies (clones 25E9 and A9E8) against human Siglec-15 or crosslinked using anti-mouse IgG F(ab’)_2_ secondary antibody. Negative controls were incubations with the isotype-matched IgG followed by anti-mouse IgG F(ab’)_2_. (**B**) Mock-transfected THP-1 and Siglec-15-expressing cells were incubated with human IgG1 KO monoclonal antibody (clone 25E09) against human Siglec-15 or crosslinked using anti-human IgG F(ab’)_2_ secondary antibody. Negative controls were incubations with isotype-matched human IgG1 followed by anti-human IgG F(ab’)_2_. For ERK and SYK inhibition, cells were pretreated with PD184352 (2 μg/mL) and BI1002494 (10 μg/mL) for 1 h before cross-linking Siglec-15 with antibodies. Cells were fixed with paraformaldehyde, permeabilized and stained for pERK1/2 (T202/Y204) antibody (1:50 dilution). Results are plotted as median fluorescence intensities ± SEM from 3 independent experiments, normalized to the untreated control. ^*^, P < 0.05; ^**^, P < 0.01; ^***^, P < 0.005; ^****^, P < 0.001; ns, not significant.

To determine whether cross-linking Siglec-15 on monocytic cells with antibodies enhanced TGF-β levels, THP-1 mock- and Siglec-15-transfected cells were incubated with two monoclonal antibodies against Siglec-15, 25E9 and A9E8, followed by cross-linking with a secondary antibody. Consistent with the co-culture studies, cross-linking Siglec-15 on THP-1 cells did not increase TGF-β secretion compared to the isotype control antibody (Figure 5D). These results suggest that Siglec-15-induced SYK signaling does not enhance TGF-β secretion. SYK signaling, however, triggers activation of other kinases including ERK1/2 and p38 MAPK, implicated in regulating inflammation in macrophages (Xiao et al. 2002). Therefore, we explored whether cross-linking Siglec-15 triggered activation of ERK and p38 MAPK in THP-1 cells expressing Siglec-15. Cross-linking Siglec-15 on THP-1 cells expressing Siglec-15 with anti-Siglec-15 whole mouse IgG1 antibodies stimulated phosphorylation of ERK1/2 (at T202/Y204) unlike the isotype control antibody, while this was inhibited when cells were pretreated with the specific MEK1/2 inhibitor PD184352 or the SYK inhibitor BI1002494 (Figure 6A and Supplementary Figure S6). Moreover, the anti-Siglec-15 human IgG KO antibody (L234A/L235A mutant), 25E09, which no longer binds Fcγ receptors also enhanced ERK phosphorylation (Figure 6B and Supplementary Figure S7) indicating that ERK activation induced by the antibodies is independent of Fc receptor engagement. These results suggest that ERK is activated downstream of Siglec-15/SYK signaling. In contrast to ERK activation, we failed to see increased phosphorylation of p38 MAPK following cross-linking of Siglec-15 in THP-1 cells (Supplementary Figure S8).

## Discussion

Siglec-15 is considered to be evolutionarily one of the most ancient Siglecs and has been mainly studied for its role in osteoclast differentiation and as a potential therapeutic target in osteoporosis (Ishida-Kitagawa et al. 2012; Hiruma et al. 2013; Stuible et al. 2014; Sato et al. 2018). Recent studies have also highlighted an important role of Siglec-15 in modulating the tumor microenvironment and favoring tumor suppression (Takamiya et al. 2013; Wang et al. 2019). This has been proposed to occur in 2 ways, either by sTn-dependent modulation of TGF-β production by Siglec-15-expressing macrophages (Takamiya et al. 2013), or by suppressing cytotoxic CD8 T cell proliferation via interactions with Siglec-15 expressed on macrophages or tumor cells (Wang et al. 2019). In this study, we have revisited the interplay between sTn and Siglec-15 in cancer immunosuppression and identified several sialylated glycan ligands of Siglec-15 other than sTn. Siglec-15-Fc multimers bound sialylated ligands on most lung and breast carcinoma cells with differential avidity. Mutation of the conserved “essential arginine” Arg143 that is critical for interaction with sialic acid to alanine and sialidase pretreatment of cancer cells resulted in significant reduction of Siglec-15 binding. Surprisingly, however, sTn expression was not detected on cancer cell lines that bound Siglec-15 and sTn overexpression in a lung cancer cell line expressing ST6GalNAc-I enzyme did not increase Siglec-15-Fc binding. Moreover, monocytic THP-1 cells overexpressing human Siglec-15 did not bind sTn-PAA-biotin sugar (data not shown). Together, these results suggest that there is a disconnect between sTn expression and Siglec-15 binding which is in contrast to previous findings (Takamiya et al. 2013).

The results of the glycan array showed clearly that Siglec-15 can bind sTn antigen and are therefore consistent with the ELISA studies reported by Angata *et al*. (Angata et al. 2007). A previous report showed that Siglec-15 could recognize both α2,6- and α2,3-linked sialic acid in the context of synthetic high affinity sialic acid analogues (Briard et al. 2018) and this is consistent with our glycan array data using natural sialosides. A new finding here is that Siglec-15 showed higher avidity binding to sialylated glycan structures, DSMFLNH (disialyl monofucosyllacto-*N*-hexaose), LSTb (sialyllacto-*N*-tetraose b) and DSLNT (disialyllacto-*N*-tetraose) which contain sialic acid α2,6-linked to an internal GlcNAc. A similar preference for this motif, as present in the milk sugar LSTb, was noted for Siglec-7 (Yamaji et al. 2002). We propose that the Siglec-15 ligands expressed on the lung and breast cancer cell lines may have structural features similar those of the glycans identified by our microarray analyses. It was shown previously that ST6GalNAcVI is the enzyme required for α2,6-sialylation of GlcNAc in disialyl Lewis a and is upregulated in colon cancer cell lines (Tsuchida et al. 2003). It will be interesting in the future to investigate the role of this enzyme in generation of Siglec-15 ligands on cancer cells. A comprehensive glycomics approach could be used to identify the potential sialoglycan ligands of Siglec-15 in these cancer cell lines (Chik et al. 2014). Another approach is to identify glycoprotein counter-receptors on cancer cells for Siglec-15 (Chang et al. 2017) and characterize their glycan ligands.

Cross-linking Siglec-15 with sialylated ligands activates ITAM signaling via DAP12 which is mediated by recruitment and activation of SYK tyrosine kinase (Ishida-Kitagawa et al. 2012; Angata 2020). Cross-linking macrophage-expressed Siglec-15 by sTn on cancer cells has been shown to enhance TGF-β secretion (Takamiya et al. 2013). This is at odds with the findings presented here, where there was no increase in TGF-β secretion upon co-culturing cancer cell lines (with and without sTn expression) with Siglec-15 over-expressing THP-1 monocytic cells. In co-culture models, the observed effects could result from engagement of multiple receptors on the surface of immune cells. This was the rationale for our testing the effect of cross-linking Siglec-15 using monoclonal antibodies as surrogate ligands. Cross-linking Siglec-15 on THP-1 cells with antibodies, however, did not enhance TGF-β secretion. SYK signaling is mediated by various intermediary kinases such as AKT, JNK (Yi et al. 2014), ERK (Eliopoulos et al. 2006, Parsa et al. 2008), p38 MAPK (Zhang et al. 2009) and PKC (Takada and Aggarwal 2004). We demonstrated by flow cytometry that cross-linking Siglec-15 on THP-1 cells triggers phosphorylation of ERK, but not p38 MAPK. Further studies are required to understand the downstream effects of Siglec-15/SYK/ERK signaling cascade.

Overall, our findings suggest that sTn, a proposed ligand of Siglec-15, is not detectably expressed on the cancer cell lines tested and that forced sTn expression does not lead to an increase in Siglec-15 binding at the cell surface. However, using structurally diverse glycan arrays, we have identified several sialylated structures that are well recognized by Siglec-15, some of which may be structurally similar to natural ligands for Siglec-15 expressed on cancer cells. From the present study it seems likely that sialylated ligands other than sTn play important roles in the signaling functions of Siglec-15, for example in osteoclast activation and suppression of CD8 T cell proliferation.

## Materials and methods

### Materials

Human Siglec-15 wildtype and R134A mutant were expressed as Fc fusion proteins by cloning the cDNAs of the extracellular domains fused to human IgG1 Fc region into pcDNA3 and transient transfection of CHO cells. Monoclonal antibodies against human Siglec-15, 25E9 (WO2011041894, Alethia Biotherapeutics) and A9E8 (WO2013034660, MedImmune) were produced by transient co-transfection of HEK293 cells with pIRES-DHFR containing the heavy chain cDNA (either murine IgG1 (25E9, A9E8) or huIgG1KO with Fc modifications to silence effector function (25E09; LALA mutant)) and pcDNA3 containing the light chain cDNA. Antibodies and Fc fusion proteins were purified by affinity chromatography on protein A/G beads. The SYK inhibitor BI1002494 (Lamb et al. 2016) was from Boehringer Ingelheim (www.opnme.com) and the MAPK inhibitor PD184352 was from the Division of Signal Transduction and Therapy (DSTT), University of Dundee. FetalClone II serum and human Fc block were from ThermoFisher Scientific. Sialidase from *Vibrio cholerae* and FITC-conjugated goat anti-human IgG Fc were from Sigma (Dorset, UK). Biotinylated MAL II was from Vector Laboratories (Peterborough, UK). Human and mouse isotype controls were from Abcam (Cambridge, UK). Anti-mouse and anti-human IgG F(ab’)2 antibodies were from Stratech (UK). Anti-sialyl Tn antibody (HB-sTn clone 3F1) was from SBH Sciences (USA). FITC conjugated Streptavidin and PE- and APC-conjugated anti-mouse IgG were from Biolegend (London, UK).

### Cell culture

All cell lines were from American Tissue Culture Collection (ATCC). HCC44, HCC827, NCI-H460, NCI-H292, NCI-H2405, MDA-MB-231, T47D, BT549, U937 and THP-1 were cultured in RPMI GlutaMAX medium supplemented with 10% (v/v) fetal bovine serum (FBS) and 1% penicillin/streptomycin. CAL120 and A549 were cultured in DMEM medium supplemented with 10% (v/v) fetal bovine serum (FBS), 2 mM L-glutamine and 1% penicillin/streptomycin. Lung cancer cell line SKMES1 was cultured in EMEM containing 10% (v/v) fetal bovine serum (FBS), 2 mM L-glutamine and 1% penicillin/streptomycin.

### Sialidase treatment of cells

Adherent cells were harvested using PBS containing 1 mM EDTA. Cells were washed twice and resuspended in RPMI medium containing 0.1 mU/mL of *V. cholerae* sialidase for 1 h at 37°C, followed by washing twice with PBS containing 1% FetalClone II serum.

### Generation of stable cell lines expressing Siglec-15 and ST6GalNAc-I

Human Siglec-15 lentiviral construct was generated by inserting human full-length Siglec-15 cDNA into pCDH-EF1-MCS-(PGK-GFP-T2A-Puro) vector. For generating the lentiviral particles, 293TN Producer Cells (System Biosciences, www.systembio.com) were cotransfected with the lentiviral expression vector and the pPACKH1 Lentiviral Packaging Plasmid Mix (System Biosciences) according to the manufacturer’s protocol. The lentiviral particles were harvested and concentrated 10-fold using PEG-itTM Virus Precipitation Solution as described by System Biosciences.

Human monocytic cell lines THP-1 and U937 (1 × 10^6^) were infected with 100 μL of lentiviral particles per well and 5 μg/mL polybrene by spinoculation. 48 h posttransfection, cells were selected with 2 μg/mL puromycin for 10 days. Puromycin resistant clones were then expanded and analyzed for GFP and Siglec-15 expression. H460 cells overexpressing sTn were generated by inserting the cDNA coding for full length ST6GalNAc-I into pIRES-puro via EcoRV and BamHI and expanding puromycin-resistant clones after transfection using lipofectamine.

### Flow cytometry analyses

#### Siglec-15-Fc Multimer Staining

Immune complexes were prepared by mixing 1 μg/mL of Siglec-15-Fc wildtype or R143A mutant and 1 μg/mL of FITC-conjugated goat anti-human IgG Fc in 100 μL phosphate-buffered saline (PBS) containing 1% FetalClone II serum (FACS buffer), for 1 h on ice. 1 × 10^6^ cancer cells were suspended in the Siglec-15-Fc immune complexes and after incubation on ice for 30 min, the nonbound immune-complexes were washed off by centrifugation and cell pellets resuspended in 500 μL of FACS buffer. Human IgG1 was used as a negative control.

#### Sialyl Tn Antibody Staining

For sTn staining, 1 × 10^6^ cells were incubated with human Fc block (2.5 μL) in 100 μL of FACS buffer for 10 min at room temperature. Cells were stained with varying concentrations of mouse monoclonal antibody against sTn (3.3 μg/mL, 10 μg/mL and 30 μg/mL). Unbound antibodies were washed off by centrifugation, and cell pellets were resuspended in PE or APC- conjugated anti-mouse IgG antibody in 100 μL of FACS buffer and incubated for 30 min on ice. Cells were centrifuged and resuspended in 500 μL of FACS buffer. Mouse isotype control was used as the negative control.

#### Intracellular Phosphoprotein Staining

THP-1 mock-transfected and Siglec-15-expressing cells were incubated with anti-human Siglec-15 antibodies (whole mouse IgG1 and human IgG1 KO) for 1 h on ice and crosslinked with anti-mouse/human IgG F(ab’)_2_ for 30 min at 37°C. Mouse/human IgG1 antibodies were used as negative controls. For inhibition experiments, THP-1 cells were pretreated with 2 μM PD184352 or 10 μM BI1002494 for 1 h at 37°C before cross-linking with anti-Siglec-15 antibodies. The cells were fixed by adding ice-cold 4% (v/v) paraformaldehyde and incubating for 15 min at room temperature. The cells were washed with cold FACS buffer and permeabilized with ice-cold 90% methanol/distilled water, added slowly while vortexing to prevent cell clumping, for 30 min on ice. The cells were washed twice with excess FACS buffer to remove the methanol before incubating first with 1:50 Fc block in FACS buffer and then with phospho-protein antibodies (1:50 p-ERK1/2 T202/Y204 or 1:50 p-p38 MAPK T180/Y182 antibodies from Cell Signaling Technologies) in FACS buffer for 1 h at room temperature. The cells were washed twice with excess FACS buffer and incubated with anti-rabbit IgG F(ab’)_2_-AF647 conjugate detection antibody (1:1000) in FACS buffer for 30 min at room temperature in the dark. The cells were washed twice with FACS buffer, resuspended in FACS buffer, and acquired on a BD FACSCanto™ II. All flow cytometry data were analyzed using FlowJo software.

### Determination of TGF-β levels by ELISA

Cancer cell lines were grown in 6-well plates until confluent and fixed using 2% paraformaldehyde for 10 min at room temperature. Cells were washed with PBS and monocytic cells (THP-1/U937) expressing human Siglec-15 (2 × 10^6^ per well) in macrophage serum free medium were added. After 24 h, culture supernatants were centrifuged to remove cell debris and concentrated 10 times using centrifugal concentrators (10 K MWCO). The concentrated culture supernatants were analyzed for TGF-β levels using Human TGF-β1 Quantikine ELISA kit (R&D Systems) and following the manufacturer’s instructions.

### Glycan array analysis

Glycan microarray analysis was carried out using a newly constructed glycan microarray comprised of 82 sequence-defined NGL probes. Sixty NGLs were prepared from synthetic amino-terminating glycans and naturally-derived serine or threonine glyco-amino acids (Supplementary Table SI; MIRAGE document). To prepare the NGLs, the amino-terminating glycans were conjugated to a the newly synthesized aldehyde-functionalized phospholipid reagent *N*-(4-formylbenzamide)-1,2-dihexadecyl-*sn*-glycero-3-phosphoethanolamine, abbreviated as DA; they are designated DA-NGLs (Supplementary Table SII; details of preparation of DA-NGLs and validation of the microarrays are described elsewhere by W Chai and colleagues, submitted). As controls, 22 conventional NGLs and glycolipids were included. The microarrays were prepared via noncovalent immobilization on nitrocellulose-coated glass slides and analyzed with the proteins following established procedures (Liu et al. 2012) (Supplementary Table SI; MIRAGE document).

### Graphs and statistics

All of the graphs (mean ± SEM) were plotted, and statistical analyses were performed using GraphPad Prism 6.07 software. Multiple comparisons of data were performed using ANOVA followed by Tukey’s post hoc HSD test for pairwise comparison.

## Supplementary Material

Supplementary_data_cwaa048

## Abbreviations

ANOVA: analysis of variance
CSF: colony stimulating factor
DA: aldehyde-functionalized phospholipid reagent *N*-(4-formylbenzamide)-1,2-dihexadecyl-*sn*-glycero-3-phosphoethanolamine
FACS: fluorescence-activated cell sorting
ITAM: immunoreceptor tyrosine-based activation motif
MAL: *Maackia amurensis* lectin
MAPK: mitogen-activated protein kinase
NGL: neoglycolipid
PBS: phosphate-buffered saline
sTn: sialyl Tn
ST6GalNAc: N-acetylgalactosaminide α-2,6-sialyltransferase
Siglec: sialic acid-binding Ig-like lectin
SYK: spleen tyrosine kinase
Siglec-15-Fc: human Siglec-15-IgG1-Fc fusion protein.
