# Supplementary material for: Siglec-15 recognition of sialoglycans on tumor cell lines can occur independently of sialyl Tn antigen expression
Source: Glycobiology. 2020 May 30;31(1):44–54. doi: 10.1093/glycob/cwaa048 (PMC7799145; doi:10.1093/glycob/cwaa048)
Supplement: Supplementary_data_cwaa048 [file supplementary_data_cwaa048.pdf]

Supplementary data

Figure S1

A.

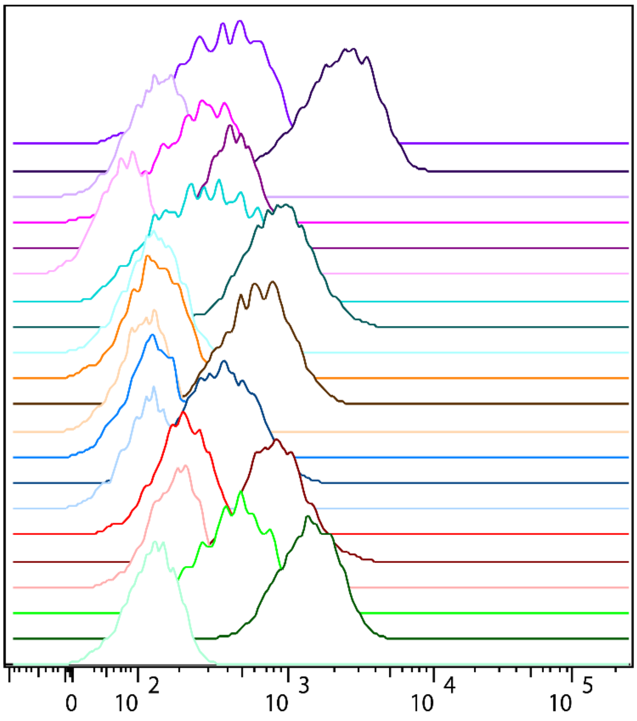

Sample	MFI
SKMES1 Sialidase Siglec-15 Fc	360
SKMES1 Siglec-15 Fc	2202
SKMES1 Anti-huIgG Fc	135
H292 Sialidase Siglec-15 Fc	248
H292 Siglec-15 Fc	402
H292 Anti-huIgG Fc	70.1
H2405 Sialidase Siglec-15 Fc	286
H2405 Siglec-15 Fc	812
H2405 Anti-huIgG Fc	121
H460 Sialidase Siglec-15 Fc	127
H460 Siglec-15 Fc	603
H460 Anti-huIgG Fc	105
HCC827 Sialidase Siglec-15 Fc	121
HCC827 Siglec-15 Fc	340
HCC827 Anti-huIgG Fc	114
HCC44 Sialidase Siglec-15 Fc	197
HCC44 Siglec-15 Fc	761
HCC44 Anti-huIgG Fc	175
A549 Sialidase Siglec-15 Fc	427
A549 Siglec-15 Fc	1323
A549 Anti-huIgG Fc	122

B.

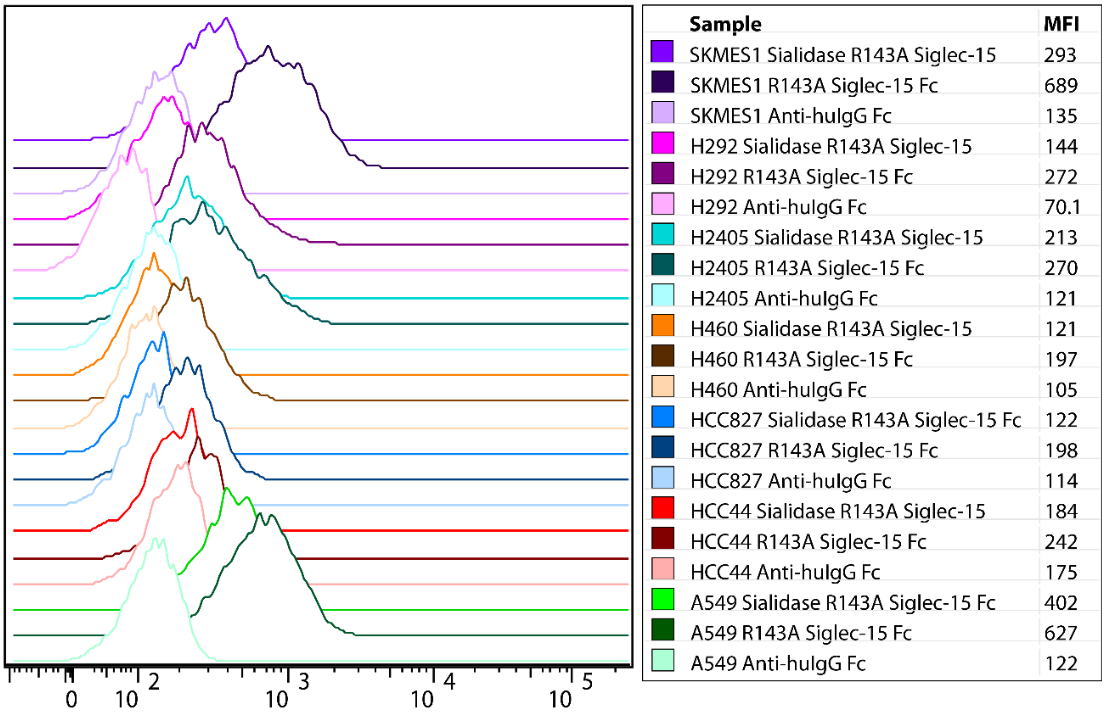

Sample	MFI
SKMES1 Sialidase R143A Siglec-15	293
SKMES1 R143A Siglec-15 Fc	689
SKMES1 Anti-huIgG Fc	135
H292 Sialidase R143A Siglec-15	144
H292 R143A Siglec-15 Fc	272
H292 Anti-huIgG Fc	70.1
H2405 Sialidase R143A Siglec-15	213
H2405 R143A Siglec-15 Fc	270
H2405 Anti-huIgG Fc	121
H460 Sialidase R143A Siglec-15	121
H460 R143A Siglec-15 Fc	197
H460 Anti-huIgG Fc	105
HCC827 Sialidase R143A Siglec-15 Fc	122
HCC827 R143A Siglec-15 Fc	198
HCC827 Anti-huIgG Fc	114
HCC44 Sialidase R143A Siglec-15	184
HCC44 R143A Siglec-15 Fc	242
HCC44 Anti-huIgG Fc	175
A549 Sialidase R143A Siglec-15 Fc	402
A549 R143A Siglec-15 Fc	627
A549 Anti-huIgG Fc	122

C.

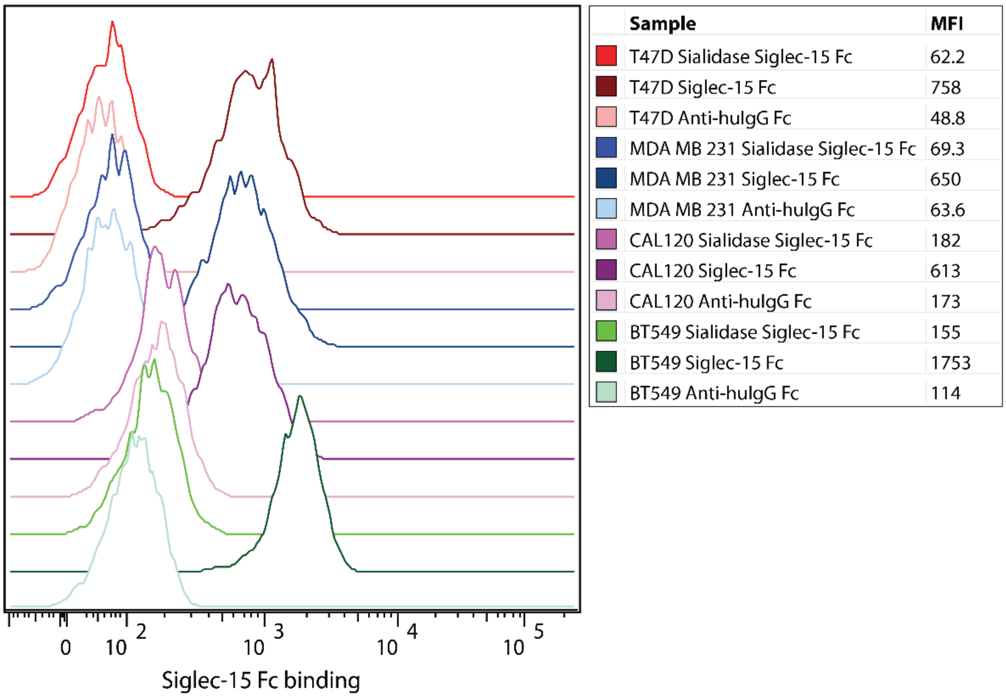

D.

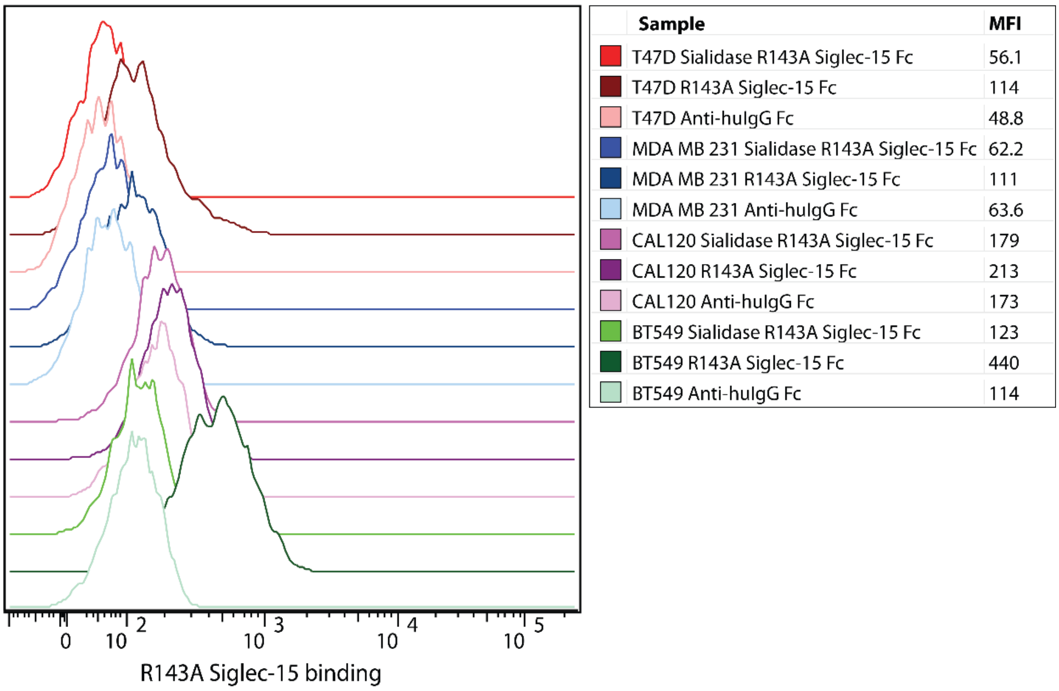

Supplementary Figure S1. Binding of Lung cancer cell lines were analysed for binding to recombinant Siglec-15-Fc wildtype **(A)** or R143A mutant **(B)** (each at 1 µg/ml) precomplexed with anti-human IgG-Fc FITC (1 µg/ml). Breast cancer cell lines were analysed for binding to Siglec-15-Fc wildtype **(C)** or R143A mutant **(D)** (each at 1 µg/ml) precomplexed with anti-human IgG Fc-FITC (1 µg/ml). Cells were pretreated with sialidase to determine whether Siglec-15 binding is sialic acid-dependent. Histograms are representative of three independent experiments.

Figure S2

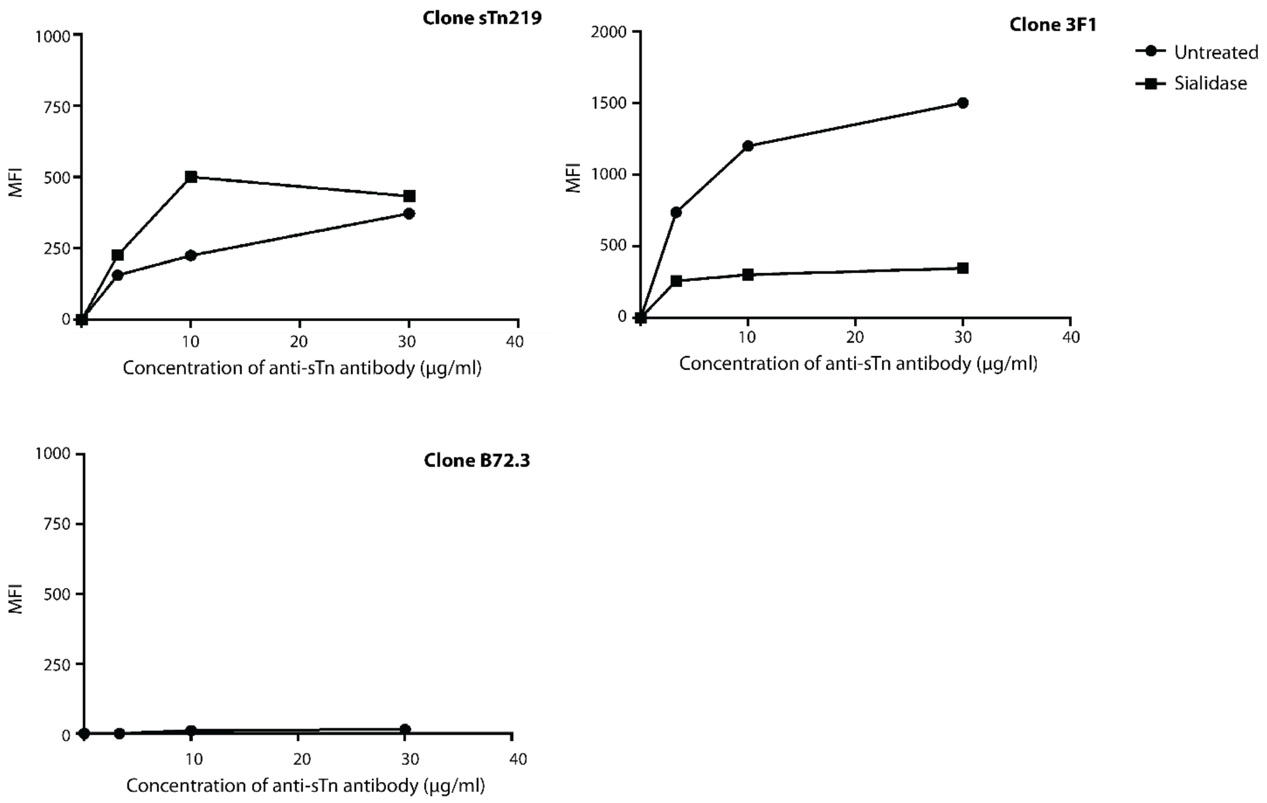

Supplementary Figure S2. Three different clones of anti-sTn antibodies were incubated with K562 cells at increasing concentrations (0, 3.3, 10 and 30 µg/ml) and tested for binding using PE-conjugated anti-mouse IgG secondary antibody. Sialidase-treated K562 cells were used as the negative control. MFI values were plotted against concentration of the anti-sTn antibody. Representative of two independent experiments.

Figure S3

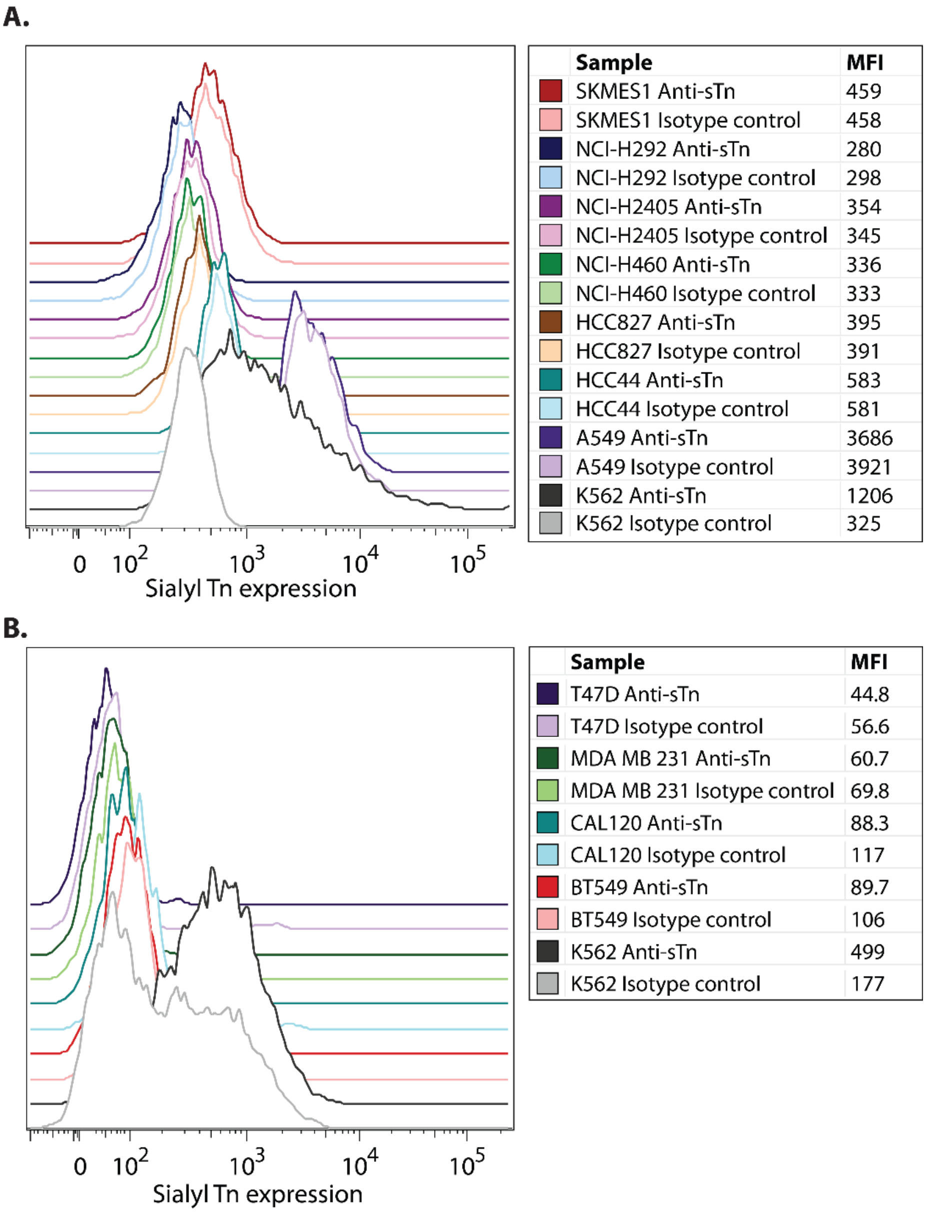

A.

Sample	MFI
SKMES1 Anti-sTn	459
SKMES1 Isotype control	458
NCI-H292 Anti-sTn	280
NCI-H292 Isotype control	298
NCI-H2405 Anti-sTn	354
NCI-H2405 Isotype control	345
NCI-H460 Anti-sTn	336
NCI-H460 Isotype control	333
HCC827 Anti-sTn	395
HCC827 Isotype control	391
HCC44 Anti-sTn	583
HCC44 Isotype control	581
A549 Anti-sTn	3686
A549 Isotype control	3921
K562 Anti-sTn	1206
K562 Isotype control	325

B.

Sample	MFI
T47D Anti-sTn	44.8
T47D Isotype control	56.6
MDA MB 231 Anti-sTn	60.7
MDA MB 231 Isotype control	69.8
CAL120 Anti-sTn	88.3
CAL120 Isotype control	117
BT549 Anti-sTn	89.7
BT549 Isotype control	106
K562 Anti-sTn	499
K562 Isotype control	177

Supplementary Figure S3. STn expression on lung **(A)** and breast **(B)** carcinoma cell lines was analysed by incubating the cells with anti-sTn antibody clone 3F1 followed by APC-conjugated anti-mouse IgG secondary antibody. K562 cells were used as a positive control and isotype-matched mouse IgG was used as a negative control. Histograms are representative of three independent experiments.

Figure S4

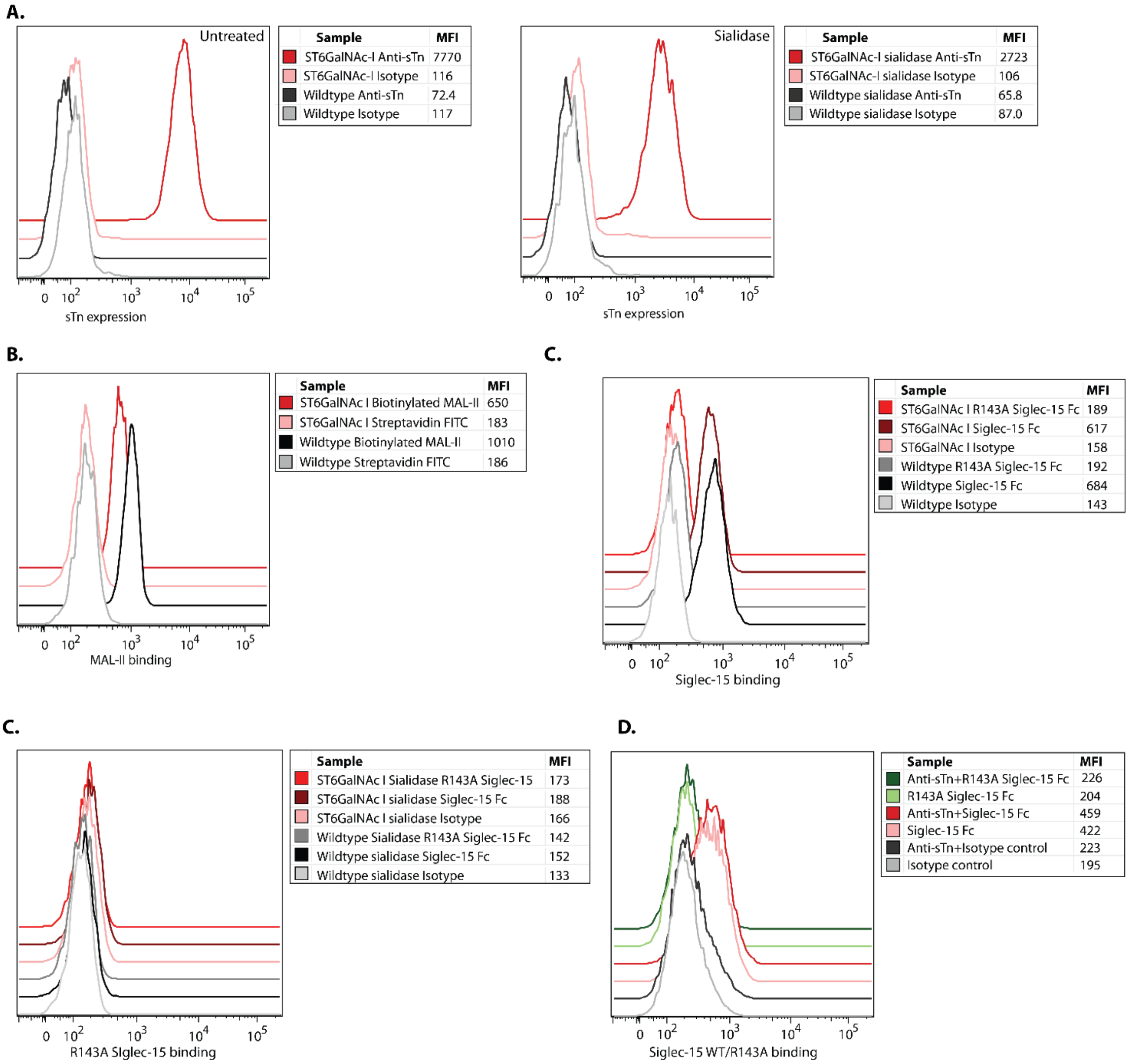

Supplementary Figure S4. (A) H460 wildtype and ST6GalNAc-I transferase-expressing cells, pretreated without or with sialidase, were tested for sTn expression using anti-sTn antibody (clone 3F1) followed by PE-conjugated anti-mouse IgG secondary antibody. **(B)** H460 cells were tested for expression of α2-3-linked sialic acids using biotinylated MAL-II (2 µg/ml) followed by streptavidin-FITC. **(C)** H460 cells with or without pre-treatment with sialidase were incubated with Siglec-15-Fc wildtype or R143A mutant precomplexed with anti-human IgG Fc-FITC. **(D)** sTn expressed on the surface of K562 cells was blocked with anti-sTn antibody (30 µg/ml) followed by Siglec-15-Fc wildtype or R143A mutant precomplexed with anti-human IgG Fc-FITC. Human isotype-matched IgG precomplexed with anti-IgG Fc-FITC was used as negative control. Histograms are representative of three independent experiments.

Figure S5

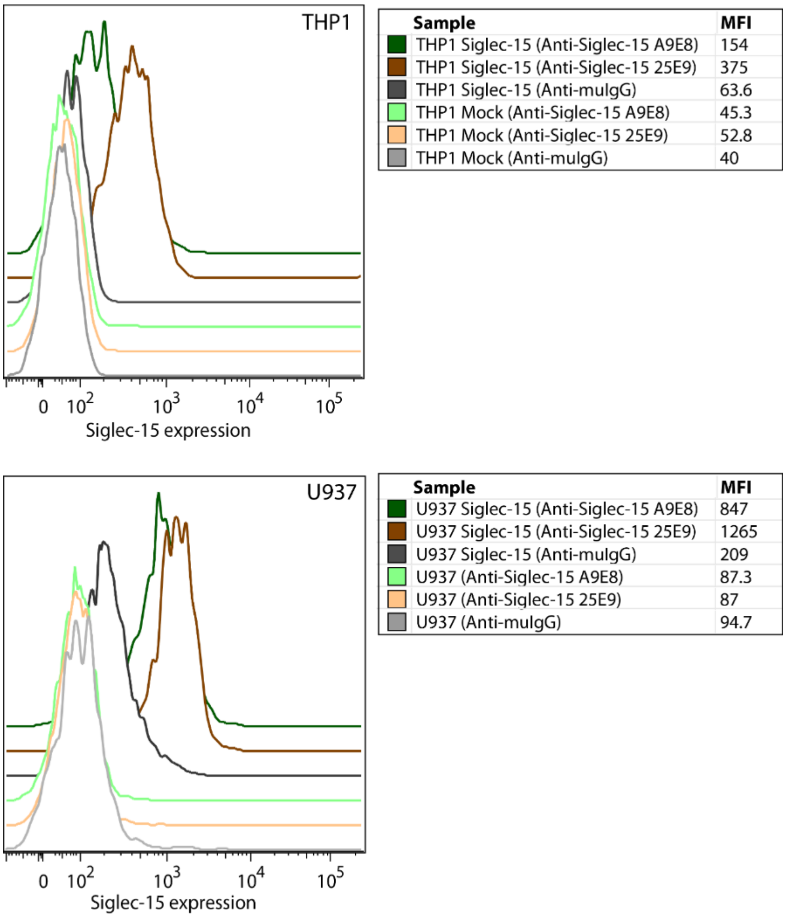

Supplementary Figure S5. Human monocytic cell lines THP-1 and U937 stably expressing human Siglec-15 were generated using the retroviral system. Expression of Siglec-15 on these cell lines was analysed by using two mouse monoclonal antibodies (clones 25E9 and A9E8) against Siglec-15 followed by anti-mouse IgG conjugated to PE.

Figure S6

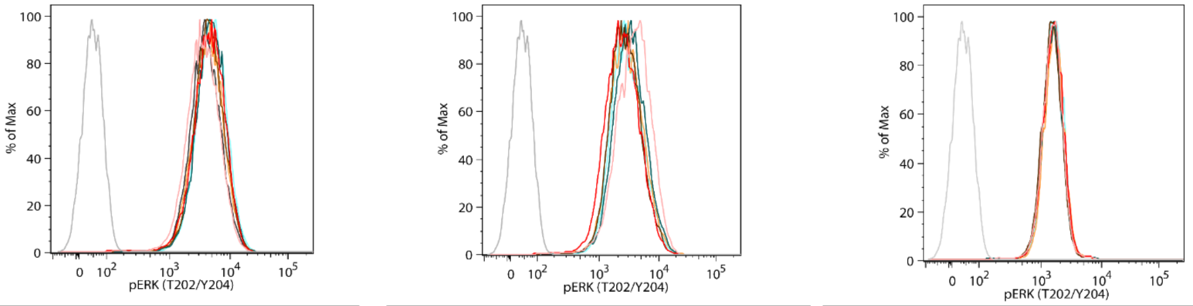

Sample	Count	Median:APC-A
THP1 Mock Unstained	10587	26.3
Untreated	10313	3110
Isotype control+Anti-mulgG	9948	4039
Anti-Siglec15 (25E9)+Anti-mulgG	10393	3882
Anti-Siglec15 (A9E8)+Anti-mulgG	10806	4213
Anti-Siglec15 (25E9)	10292	3806
Anti-Siglec15 (A9E8)	11507	4329
Anti-mulgG	10824	3415

Sample	Count	Median:APC-A
THP1 Mock Unstained	10587	26.3
iSYK+Untreated	10043	3483
iSYK+Isotype control+Anti-mulgG	9222	2149
iSYK+Anti-Siglec15 (25E9)+Anti-mulgG	9958	2386
iSYK+Anti-Siglec15 (A9E8)+Anti-mulgG	8551	2860
iSYK+Anti-Siglec15 (25E9)	11824	2500
iSYK+Anti-Siglec15 (A9E8)	9836	2537

Sample	Count	Median:APC-A
THP1 Mock Unstained	10587	26.3
PD184352+Untreated	9636	1415
PD184352+Isotype+Anti-mulgG	9662	1453
PD184352+Anti-Sig15(25E9)+Anti-mulgG	9854	1368
PD184352+Anti-Sig15(A9E8)+Anti-mulgG	10232	1408
PD184352+Anti-Sig15(25E9)	9871	1499
PD184352+Anti-Sig15(A9E8)	9641	1470

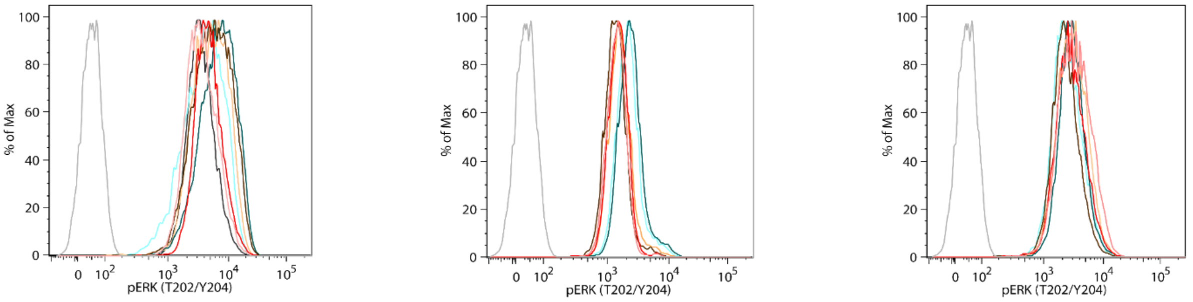

Sample	Count	Median:APC-A
THP1 Siglec-15 Unstained	11531	28.9
Untreated	10322	2975
Isotype+Anti-mulgG	9949	3970
Anti-Siglec-15 (25E9)+Anti-mulgG	10900	4852
Anti-Siglec-15 (A9E8)+Anti-mulgG	10997	6207
Anti-Siglec-15 (25E9)	8386	4438
Anti-Siglec-15 (A9E8)	11198	3543
Anti-mulgG	10017	2960

Sample	Count	Median:APC-A
THP1 Siglec-15 Unstained	11531	28.9
PD184352+Untreated	11477	1378
PD184352+Isotype+Anti-mulgG	10157	1467
PD184352+Anti-Sig15 (25E9)+Anti-mulgG	12384	1317
PD184352+Anti-Sig15 (A9E8)+Anti-mulgG	11928	2267
PD184352+Anti-Sig15 (25E9)	12140	1539
PD184352+Anti-Sig15 (A9E8)	11775	2062

Sample	Count	Median:APC-A
THP1 Siglec-15 Unstained	11531	28.9
iSYK+Untreated	11542	2903
iSYK+Isotype+Anti-mulgG	9957	2445
iSYK+Anti-Siglec 15 (25E9)+Anti-mulgG	10680	2008
iSYK+Anti-Siglec 15 (A9E8)+Anti-mulgG	11115	2632
iSYK+Anti-Siglec 15 (25E9)	9584	2593
iSYK+Anti-Siglec 15 (A9E8)	9789	2113

Supplementary Figure S6. THP-1 mock-transfected and Siglec-15-expressing cells, with or without pre-treatment with PD184352 (2 µM) or iSYK/BI1002494 (10 µM), were incubated with mouse anti-human Siglec-15 antibodies (6 µg/ml) (clones 25E9 and A9E8) followed by cross-linking with anti-mouse IgG F(ab')2 secondary antibody (3 µg/ml). Cells were fixed, permeabilised and stained using rabbit anti-ERK pT202/Y204 phosphospecific antibody followed by anti-rabbit IgG F(ab')$_2$-AF647 secondary antibody. Isotype-matched mouse antibody was used as the negative control. Histograms are representative of three independent experiments.

Figure S7

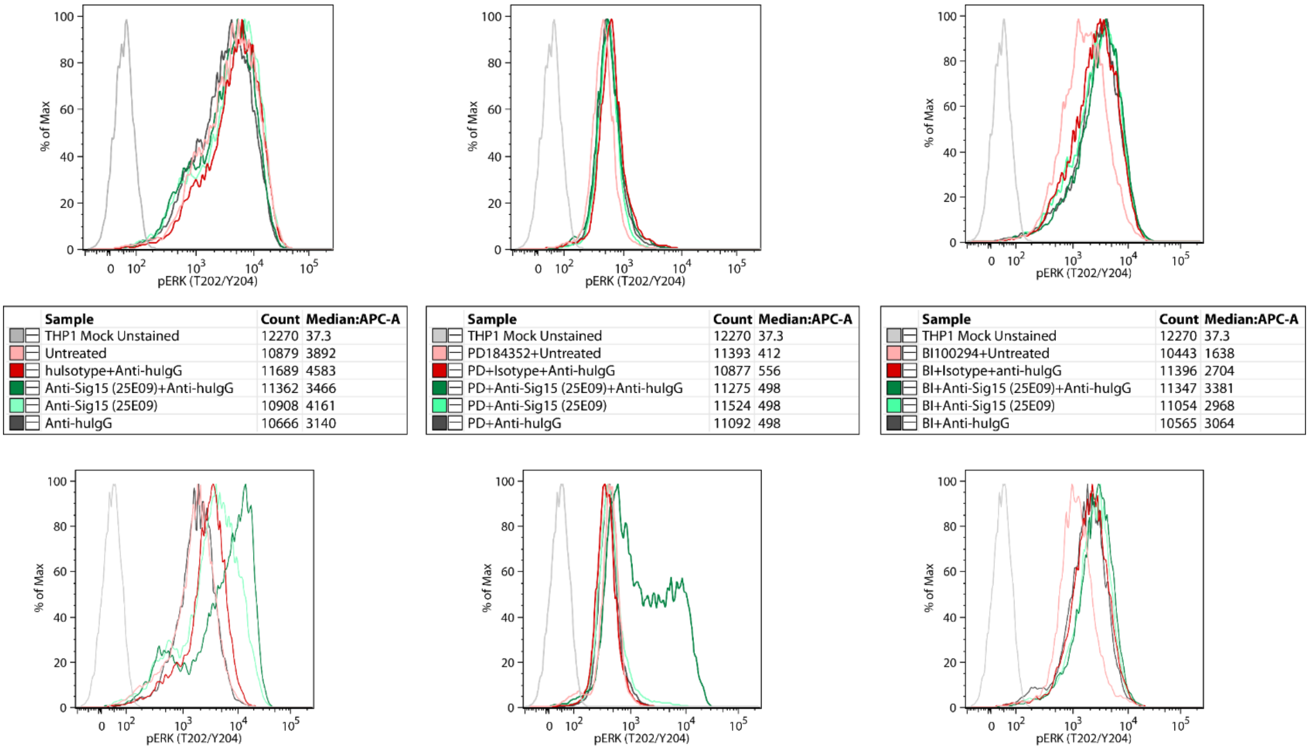

Sample	Count	Median:APC-A
THP1 Mock Unstained	12270	37.3
Untreated	10879	3892
huIsotype+Anti-huIgG	11689	4583
Anti-Sig15 (25E09)+Anti-huIgG	11362	3466
Anti-Sig15 (25E09)	10908	4161
Anti-huIgG	10666	3140

Sample	Count	Median:APC-A
THP1 Mock Unstained	12270	37.3
PD184352+Untreated	11393	412
PD+Isotype+Anti-huIgG	10877	556
PD+Anti-Sig15 (25E09)+Anti-huIgG	11275	498
PD+Anti-Sig15 (25E09)	11524	498
PD+Anti-huIgG	11092	498

Sample	Count	Median:APC-A
THP1 Mock Unstained	12270	37.3
BI100294+Untreated	10443	1638
BI+Isotype+anti-huIgG	11396	2704
BI+Anti-Sig15 (25E09)+Anti-huIgG	11347	3381
BI+Anti-Sig15 (25E09)	11054	2968
BI+Anti-huIgG	10565	3064

Sample	Count	Median:APC-A
THP1 Sig-15 Unstained	11350	39.8
Untreated	11877	1749
Isotype+Anti-huIgG	13029	2953
Anti-Sig15 (25E09)+Anti-huIgG	12314	6841
Anti-Sig15 (25E09)	12023	3778
Anti-huIgG	11250	1804

Sample	Count	Median:APC-A
THP1 Sig15 Unstained	11350	39.8
PD184352+Untreated	11970	435
PD+Isotype+Anti-huIgG	13059	376
PD+Anti-Sig15 (25E09)+anti-huIgG	12842	1289
PD+Anti-Sig15 (25E09)	12796	427
PD+Anti-huIgG	13154	413

Sample	Count	Median:APC-A
THP1 Sig15 Unstained	11350	39.8
BI100294+Untreated	12355	1142
BI+Isotype+Anti-huIgG	12460	2092
BI+Anti-Sig15 (25E09)+Anti-huIgG	12899	2645
Anti-Sig15 (25E09)	11205	2422
Anti-huIgG	12519	1903

Supplementary Figure S7. THP-1 mock-transfected and Siglec-15-expressing cells, with or without pre-treatment with PD184352 (2 µM) or iSYK/BI1002494 (10 µM), were incubated with anti-Siglec-15 human IgG1 KO antibody (6 µg/ml) (clone 25E09) followed by cross linking with anti-human IgG F(ab')2 secondary antibody (3 µg/ml). Cells were fixed, permeabilised and stained using rabbit anti-ERK pT202/Y204 phosphospecific antibody followed by anti-rabbit IgG F(ab')$_2$-AF647 secondary antibody. Human isotype-matched antibody was used as the negative control. Histograms are representative of three independent experiments.

Figure S8

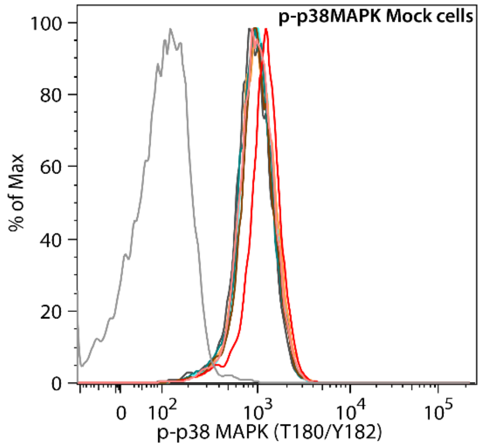

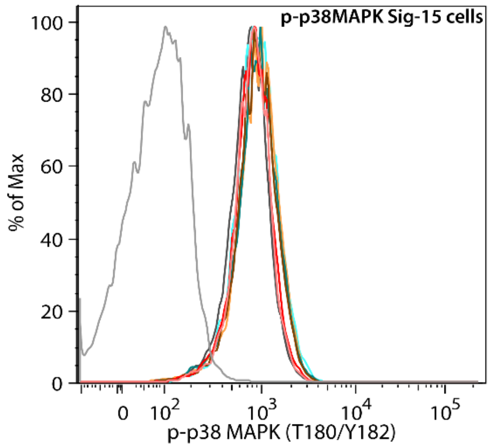

Sample	Count	MFI
THP1 Mock Unstained	5553	112
Untreated	5525	1039
Isotype+Anti-muIgG	5193	1280
Anti-Sig15 (25E9)+Anti-muIgG	5505	1039
Anti-Sig15 (A9E8)+Anti-muIgG	5014	1010
Anti-Sig15 (25E9)	5379	1049
Anti-Sig15 (A9E8)	5826	1046
Anti-muIgG	5207	959

Sample	Count	MFI
THP1 Siglec-15 Unstained	7860	87.8
Untreated	8434	843
Isotype+Anti-muIgG	8198	847
Anti-Sig15 (25E9)+Anti-muIgG	8264	926
Anti-Sig15 (A9E8)+Anti-muIgG	8081	934
Anti-Sig15 (25E9)	8104	963
Anti-Sig15 (A9E8)	8659	924
Anti-muIgG	8395	784

Supplementary Figure S8. THP1 mock-transfected and Siglec-15-expressing cells were incubated with mouse anti-human Siglec-15 antibodies (6 µg/ml) (clones 25E9 and A9E8) followed by cross linking with anti-mouse IgG F(ab')2 secondary antibody (3 µg/ml). Cells were fixed, permeabilised and stained using rabbit anti-p38 MAPK pT180/Y182 phosphospecific antibody followed by anti-rabbit IgG F(ab')$_2$-AF647 secondary antibody. Isotype-matched mouse IgG was used as the negative control. Histograms are representative of two independent experiments.

Supplementary Table SI. Glycan microarray document based on MIRAGE Glycan Microarray Guidelines (doi:10.3762/mirage.3)

Classification	Guidelines
**1. Sample: Glycan Binding Sample**	
Description of Sample	Sample names:<br>Human Siglec-15 wildtype (hSiglec-15 WT) and Arg134Ala mutant (hSiglec-15 ArgR143Ala) are recombinant and were expressed as Fc-fusion proteins as described in materials methods section in the main text.<br>Anti-sialyl Tn monoclonal antibody (anti-STn 3F1-IgG) was from SBH Sciences (USA).
Sample modifications	Not relevant.
Assay protocol	Assay procedures were according established protocols (Liu et al., Methods Mol Biol. 2012)(Liu et al. 2012). In brief, the arrayed slides were blocked with 2% w/v BSA in 100 mM Phosphate Buffer Saline pH 7.4 (2%BSA/PBS). The Siglec-15 chimeras were analysed as multimers at 2 µg/ml, prepared by pre-complexing with the biotinylated goat anti-human IgG (VECTOR, BA300) at 1:1 ratio (by weight). The Siglec-15-antibody complex was prepared by pre-incubating the protein and antibody, diluted in the final required volume of blocking buffer, for 1h at 4°C. The anti-sialyl Tn antibody 3F1 was analysed at 50µg/ml prepared in binding buffer (1% BSA/PBS), followed by incubation (1h) with 10 µg/ml biotinylated anti-mouse IgG (SIGMA, B-7264) .
**2. Glycan Library**	
Glycan description for defined glycans	Sixty amino-terminating glycans (aminoalkyl-, phenyl- or glycine-terminating, or natural serine or threonine terminating amino acids) were used to prepare neoglycolipids (NGLs). These are listed and referenced below, together with 22 reducing oligosaccharides or glycosylceramides used as reference glycans.

Probe Designation	Pos.[a]	Sequence[b]	Glycan sources
**Amino-terminating glycans**			
Lac-C2-N	1	Galβ-4Glcβ-O-C2-NH2	W Chai and colleagues (submitted)
2'FL-Gly	2	Fucα-2Galβ-4Glcβ-NH-Gly	Human milk (Likhosherstov et al. 2012)
B-tri-C3-N	3	Galα-3Galβ-O-C3-NH2<br>| Fucα-2	Elicityl
3'SA-Lac-C2-N	4	NeuAcα-3Galβ-4Glcβ-O-C2-NH2	Elicityl
3'SA-Lac-Gly	5	NeuAcα-3Galβ-4Glcβ-NH-Gly	Human milk (Likhosherstov et al. 2016)
6'SA-Lac-Gly	6	NeuAcα-6Galβ-4Glcβ-NH-Gly	Human milk (Likhosherstov et al. 2016)
LNFP I-Gly	7	Fucα-2Galβ-3GlcNAcβ-3Galβ-4Glcβ-NH-Gly	Human milk (Likhosherstov et al. 2012)
LSTb-Gly	8	Galβ-3GlcNAcβ-3Galβ-4Glcβ-NH-Gly<br>| NeuAcα-6	Human milk (Likhosherstov et al. 2016)
DSLNT-Gly	9	NeuAcα-3Galß-3GlcNAcß-3Galß-4Glcß-NH-Gly<br>| NeuAcα-6	Human milk (Likhosherstov et al. 2016)
DSMFLNH-Gly	10	Galß-4GlcNAcß-6<br>| | Fucα-3 Galß-4Glcβ-NH-Gly<br>| NeuAcα-3Galß-3GlcNAcß-3<br>| NeuAcα-6	Human milk (Likhosherstov et al. 2016)
ChitoBiose-Gly	11	GlcNAcβ-4GlcNAcβ-NH-Gly	Chitin (Nishimura et al. 1989)
Fuc(3)GlcNAc-Gly	12	GlcNAcβ-NH-Gly<br>| Fucα-3	Chemical synthesis (Likhosherstov et al. 2015)

Fuc(6)GlcNAc-Gly	13	Fucα-6GlcNAcβ-NH-Gly	Chemical synthesis (Likhosherstov et al. 2015)
DFuc(3,6)GlcNAc-Gly	14	Fucα-6GlcNAcβ-NH-Gly<br>Fucα-3	Chemical synthesis (Likhosherstov et al. 2015)
Man5-Gly	15	Manα-6<br>Manα-3Manα-6<br>Manβ-4GlcNAcβ-4GlcNAcβ-NH-Gly<br>Manα-3	Chicken egg white (Piskarev et al. 1990)
Man6-Gly	16	Manα-6<br>Manα-3Manα-6<br>Manβ-4GlcNAcβ-4GlcNAcβ-NH-Gly<br>Manα-2Manα-3	Chicken egg white (Piskarev et al. 1990)
NA2-Gly	17	Galβ-4GlcNAcβ-2Manα-6<br>Manβ-4GlcNAcβ-4GlcNAcβ-NH-Gly<br>Galβ-4GlcNAcβ-2Manα-3	Chicken egg white (Piskarev et al. 1990)
NA2F-Gly	18	Galβ-4GlcNAcβ-2Manα-6 Fucα-6<br>Manβ-4GlcNAcβ-4GlcNAcβ-NH-Gly<br>Galβ-4GlcNAcβ-2Manα-3	Chicken egg white (Piskarev et al. 1990)
SM1a-C3-N	19	Galβ-3GalNAcβ-4Galβ-4Glcβ-O-C3-NH2<br>SU-3	W Chai and colleagues (submitted)
SM1a(2S)-C3-N	20	Galβ-3GalNAcβ-4Galβ-4Glcβ-O-C3-NH2<br>SU-2	W Chai and colleagues (submitted)
GalNAcα-ON	21	GalNAcα-O-NH2	Elicityl
GalNAcα-Ser	22	GalNAcα-O-Ser	Sussex Research
GalNAcα-Thr	23	GalNAcα-O-Thr	Dextra Lab
GalNAcβ-Ser	24	GalNAcβ-O-Ser	W Chai and colleagues (submitted)
GalNAcβ-Thr	25	GalNAcβ-O-Thr	W Chai and colleagues (submitted)
GlcNAcβ-Ser	26	GlcNAcβ-O-Ser	W Chai and colleagues (submitted)
GlcNAcβ-Thr	27	GlcNAcβ-O-Thr	W Chai and colleagues (submitted)
STn-Ser	28	NeuAcα-6GalNAcα-O-Ser	W Chai and colleagues (submitted)
STn-Thr	29	NeuAcα-6GalNAcα-O-Thr	W Chai and colleagues (submitted)
Core 1-Ser	30	Galβ-3GalNAcα-O-Ser	Dextra Lab
Core 1-Thr	31	Galβ-3GalNAcα-O-Thr	Sussex Research
SA1(2-3)-Core 1-Ser	32	NeuAcα-3Galβ-3GalNAcα-O-Ser	W Chai and colleagues (submitted)
SA1(2-3)-Core 1-Thr	33	NeuAcα-3Galβ-3GalNAcα-O-Thr	W Chai and colleagues (submitted)
SA1(2-6)-Core 1-Ser	34	Galβ-3GalNAcα-O-Ser<br>NeuAcα-6	Human urine (Parkkinen and Finne 1983)
SA1(2-6)-Core 1-Thr	35	Galβ-3GalNAcα-O-Thr<br>NeuAcα-6	Human urine (Parkkinen and Finne 1983)
SA2-Core 1-Ser	36	NeuAcα-3Galβ-3GalNAcα-O-Ser<br>NeuAcα-6	Human urine (Parkkinen and Finne 1983)
SA2-Core 1-Thr	37	NeuAcα-3Galβ-3GalNAcα-O-Thr<br>NeuAcα-6	Human urine (Parkkinen and Finne 1983)
Core 2-C3-N	38	Galβ-3GalNAcα-O-C3-NH2<br>GlcNAcβ-6	Nicolai Bovin
Core 2-Thr	39	Galβ-3GalNAcα-O-Thr<br>GlcNAcβ-6	Sussex Research
Core 3-Ser	40	GlcNAcβ-3GalNAcα-O-Ser	Sussex Research
Core 3-Thr	41	GlcNAcβ-3GalNAcα-O-Thr	Sussex Research
Gal-Core 3-Ser	42	Galβ-4GlcNAcβ-3GalNAcα-O-Ser	W Chai and colleagues (submitted)

Gal(3S)-Core 3-Ser	43	Gal(3S)β-4GlcNAcβ-3GalNAcα-O-Ser	W Chai and colleagues (submitted)
GalNAc-G(3S)-Core 3-Ser	44	GalNAcβ-4Gal(3S)β-4GlcNAcβ-3GalNAcα-O-Ser	W Chai and colleagues (submitted)
GSC 967-Ser	45	Galβ-3GalNAcβ-4Gal(3S)β-4GlcNAcβ-3GalNAcα-O-Ser	W Chai and colleagues (submitted)
Core 4-C3-N	46	GlcNAcβ-3GalNAcα-O-C3-NH2<br>| GlcNAcβ-6	Nicolai Bovin
Core 4-Thr	47	GlcNAcβ-3GalNAcα-O-Thr<br>| GlcNAcβ-6	Sussex Research
Man-PentaPep	48	YAT(Man)AV	Chemical synthesis (Bartels et al. 2016)
Man-HexaPep	49	CYAT(Man)AV-NMe	Chemical synthesis (Bartels et al. 2016)
Man-UndecaPep	50	Sp-TriEG-SQSLEET(Man)ISPR	Chemical synthesis (Bartels et al. 2016)
Hep-4-NAc-PhN	51	GlcNAcα-4GlcAβ-4GlcNAcα-4GlcAβ-O-Ph-NH2	Chemo-enzymatic synthesis (Jian Liu)
Hep-4-NS-PhN	52	GlcNSα-4GlcAβ-4GlcNSα-4GlcAβ-O-Ph-NH2	Chemo-enzymatic synthesis (Jian Liu)
Glc12-C5-N	53	Glcβ-3Glcβ-3Glcβ-3Glcβ-[3Glcβ-3Glcβ]$_3$-3Glcβ-3Glcβ-O-C5-NH2	Chemical synthesis (Weishaupt et al. 2013, 2017)
Glc15-C2-N	54	Glcβ-3Glcβ-3Glcβ-3Glcβ-[3Glcβ-3Glcβ]$_3$-3Glcβ-3Glcβ-3Glcβ-3Glcβ-3Glcβ-O-C2-NH2	Chemical synthesis Novartis
Glc13(B10)-C5-N	55	Glcβ-3Glcβ-3Glcβ-3Glcβ-[3Glcβ-3Glcβ]$_3$-3Glcβ-3Glcβ-O-C5-NH2<br>| Glcβ-6	Chemical synthesis (Weishaupt et al. 2013, 2017)
Gal-C2-N	56	Galβ-O-C2-NH2	W Chai and colleagues (submitted)
Gal-Ph-N	57	Galβ-O-Ph-NH2	W Chai and colleagues (submitted)
Glc-C2-N	58	Glcβ-O-C2-NH2	W Chai and colleagues (submitted)
Man-C2-N	59	Manβ-O-C2-NH2	W Chai and colleagues (submitted)
Xyl-C2-N	60	Xylβ-O-C2-NH2	W Chai and colleagues (submitted)
**Reducing glycans and glycosylceramides**			
Lac	61	Galβ-4Glc	(Palma et al. 2011; Vendele et al. 2020)
B-Tetra-T2	62	Galα-3Galβ-4GlcNAc<br>| Fucα-2	W Chai and colleagues (submitted)
NeuAcα-(3')Lac	63	NeuAcα-3Galβ-4Glc	(Palma et al. 2011; Vendele et al. 2020)
LNFP-I	64	Fucα-2Galβ-3GlcNAcβ-3Galβ-4Glc	(Palma et al. 2011; Vendele et al. 2020)
LNFP-III	65	Galβ-4GlcNAcβ-3Galβ-4Glc<br>| Fucα-3	(Palma et al. 2011; Vendele et al. 2020)
LSTb	66	Galβ-3GlcNAcβ-3Galβ-4Glc<br>| NeuAcα-6	(Palma et al. 2011; Vendele et al. 2020)
H2 (with H2+Fuc)	67	Fucα-2Galβ-4GlcNAcβ-3Galβ-4GlcNAcβ-3Galβ-4Glc(β-Cer)*	(Gao et al. 2014)
TFiLNO	68	Galβ-3GlcNAcβ-3Galβ-4GlcNAcβ-6<br>| | | Fucα-4 Fucα-3 Galβ-4Glc<br>| Galβ-3GlcNAcβ-3<br>| Fucα-4	(Palma et al. 2011; Vendele et al. 2020)
SM1a	69	Galβ-3GalNAcβ-4Galβ-4Glc(β-Cer)<br>| SU-3	(Palma et al. 2011; Vendele et al. 2020)
Asialo-GM1-Tetra	70	Galβ-3GalNAcβ-4Galβ-4Glc	(Palma et al. 2011; Vendele et al. 2020)
SB1a	71	SU-3Galβ-3GalNAcβ-4Galβ-4Glc(β-Cer)<br>| SU-3	(Palma et al. 2011; Vendele et al. 2020)
GalNAc-Ser	72	GalNAcα-Ser	(Palma et al. 2011; Vendele et al. 2020)
GalNAc-Thr	73	GalNAcα-Thr	(Palma et al. 2011; Vendele et al. 2020)
BSM-Di-A1	74	NeuGcα-6GalNAc	(Chai et al. 1992)
BSM-Di-A2	75	NeuAcα-6GalNAc	(Chai et al. 1992)
Galβ-3GalNAc	76	Galβ-3GalNAc	(Palma et al. 2011; Vendele et al. 2020)
Galβ-6GalNAc	77	Galβ-6GalNAc	(Palma et al. 2011; Vendele et al. 2020)

DST	78	NeuAcα-3Galβ-3GalNAc<br>| NeuAcα-6	(Palma et al. 2011; Vendele et al. 2020)
Curd-13	79	Glcβ-3Glcβ-3Glcβ-3Glcβ-[3Glcβ-3Glcβ]$_3$-3Glcβ-3Glcβ-3Glc*	(Palma et al. 2015)
Cello-3	80	Glcβ-4Glcβ-4Glc	(Palma et al. 2015)
GN2	81	GlcNAcβ-4GlcNAc	(Palma et al. 2011; Vendele et al. 2020)
GN6	82	GlcNAcβ-4GlcNAcβ-4GlcNAcβ-4GlcNAcβ-4GlcNAcβ-4GlcNAc*	(Vendele et al. 2020)

[a] Pos, position in the microarray with glycans sorted according to backbone type.
[b] Abbreviations of reducing terminal structures: Gly, glycine; Ph, phenyl; Ser, serine; Thr, threonine; C2, -CH_2-CH_2-; C3, -CH_2-CH_2-CH_2-; C5, -CH_2-$(CH_2)_3$-CH_2-.
*Asterisks indicate that major components are depicted.

Glycan description for undefined glycans	Not applicable.
Glycan modifications	The amino-terminating glycans were conjugated to a new aldehyde-functionalized phospholipid reagent *N*-(4-formylbenzamide)-1,2-dihexadecyl-*sn*-glycero-3-phosphoethanolamine, abbreviated to DA (W Chai and colleagues, submitted). The NGLs were designated DA-NGLs.<br>DH, NGLs, prepared from reducing oligosaccharides by reductive amination with the amino lipid, 1,2-dihexadecyl-*sn*-glycero-3-phosphoethanolamine [(DHPE) (Chai et al., Methods Enzymol. 2003)](Chai et al. 2003); AO, NGLs prepared from reducing oligosaccharides by oxime ligation with an aminooxy functionalized DHPE [(AOPE) (Liu et al., Chem. Biol. 2007)](Liu et al. 2007).
**3. Printing Surface; e.g., Microarray Slide**	
Description of surface	Nitrocellulose-coated glass microarray slides.
Manufacturer	16-pad UniSart® 3D Microarray Slide from Sartorius (Goettingen, Germany).
Custom preparation of surface	Not relevant.
Non-covalent Immobilization	The lipid-linked oligosaccharide probes were formulated as liposomes by adding carrier lipids, 1,2-dihexanoyl-*sn*-glycero-3-phosphocholine (DHPC) and cholesterol for arraying and non-covalent immobilization on nitrocellulose-coated glass slides (Liu et al., Methods Mol. Biol. 2012)(Liu et al. 2012).
**4. Arrayer (Printer)**	
Description of Arrayer	Nano-Plotter 2.1 (GeSiM, Radeberg, Germany).
Dispensing mechanism	Non-contact liquid delivery with four dispensing tips.
Glycan deposition	Approximately 0.33 nl was printed per spot.<br>Each glycan probe was printed at 2 levels (2 and 5 fmol per spot), in duplicate.
Printing conditions	The printing solutions were aqueous-based. Printing was performed at ambient temperature and relative humidity of 58%.<br>The NGL printing solutions contained 100 pmol/µl of DHPC and cholesterol (both from SIGMA) as lipid carriers in addition to the lipid-linked glycan probes. The concentrations of the NGL probes were 5 and 15 pmol/µl for the 2 and 5 fmol per spot levels, respectively.<br>The printing solutions also contained Cy3 NHS ester (GE Healthcare) at 20 ng/ml (26 fmol/µl) as a marker to monitor the printing process.
**5. Glycan Microarray with "Map"**	

Array layout	Each array slide contained 16-pad subarrays. Each pad was set up for printing 64 probes maximum, each at 2 levels in duplicate (four spots for one probe in a row); up to 256 spots (16x16) in total for each subarray. The probes were printed on multiple subarrays for parallel binding analyses.
Glycan identification and quality control	Quality control was performed with sequence-specific proteins: biotinylated plant lectins - Wheat Germ Agglutinin (WGA), *Vicia Villosa* Lectin (VVL), Peanut agglutinin (PNA), *Helix pomatia* lectin (HPA), *Ricinus Communis* agglutinin I (RCA_{120}), Concanavalin A (ConA) and *Aleuria aurantia* lectin (AAL) (Vector Laboratories); Fc-tagged-MGL (CLEC10A) (Abcam); his-tagged carbohydrate-binding modules of bacterial glycoside hydrolases (*Cm*CBM6-2, *Tm*CBM4-2); CTD110.6 – anti-O-GlcNAc (Santa Cruz) and anti-blood group H monoclonal antibodies (17-206, BRIC231) (Abcam) (W Chai and colleagues, submitted).
**6. Detector and Data Processing**	
Scanning hardware	GenePix 4300A (Molecular Devices)
Scanner settings	Scanning resolution: 10 µm / pixel;<br>Laser channel: red (scan wavelength 635nm);<br>PMT Voltages: 350<br>Scan power: Adjusted for each sample to achieve maximum signal without saturation of any single spot.
Image analysis software	GenePix® Pro 7 (Molecular Devices)
Data processing	The gpr file was entered into an in-house microarray database using software (designed by Mark Stoll, http://www.beilstein-institut.de/en/publications/proceedings/glyco-2009) for data processing. No particular normalization method or statistical analysis was used.
**7. Glycan Microarray Data Presentation**	
Data presentation	In Figure 4, the binding results are presented as 2D bar graphs with bars representing averaged mean signal of duplicate spots at 5 fmol/spot and error bars representing standard deviation. The fluorescence intensity numerical scores are depicted in **Supplementary Table S2**.
**8. Interpretation and Conclusion from Microarray Data**	
Data interpretation	No software or algorithms were used to interpret processed data.
Conclusions	Human Siglec-15 is shown to bind to sialylated glycans apart from sTn. Mutation of the conserved Arg143 that is critical for interaction with sialic acid to Ala markedly reduced but not abolished the binding. The binding of hSiglec-15 contrasts with the restricted binding observed with the 3F1-IgG antibody to the sTn antigen.<br>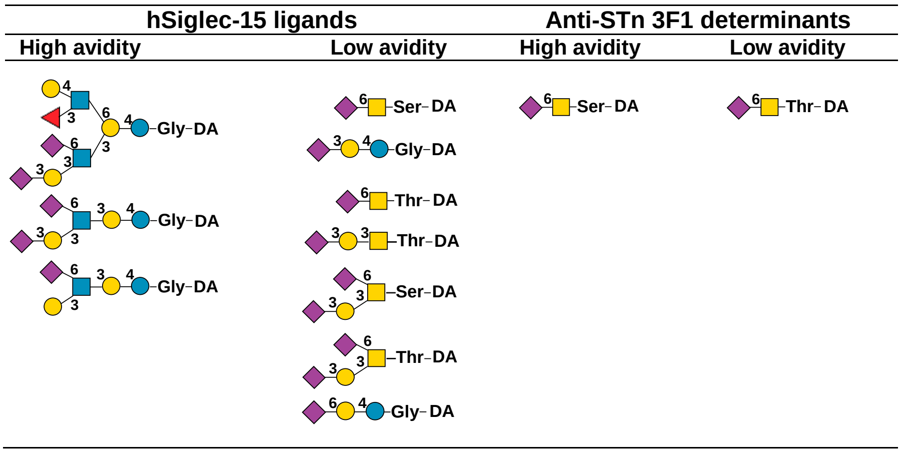

Supplementary Table SII. List of the lipid-linked glycan probes included in microarrays and the fluorescence binding intensities elicited with human Siglec-15 proteins and 3F1 antibody.

			Fluorescence intensities[3] (5fmol/spot)		
**Probe name[1]**	**Pos.**	**Probe Sequence[2]**	**hSiglec-15 WT**	**hSiglec-15 Arg143Ala**	**Anti-STn 3F1-IgG**
**DA-NGLs**					
Lac-C2-N-	1	Galβ-4Glcβ-O-C2-NX-DA	-	-	-
2'FL-Gly-	2	Fucα-2Galβ-4Glc-NH-Gly-NH-DA	-	-	-
B-tri-C3-N-	3	Galα-3Galβ-O-C3-NX-DA<br>| Fucα-2	-	-	-
3'SA-Lac-C2-N-	4	NeuAcα-3Galβ-4Glcβ-O-C3-NX-DA	-	-	-
3'SA-Lac-Gly-	5	NeuAcα-3Galβ-4Glcβ-NH-Gly-NH-DA	1 451	-	-
6'SA-Lac-Gly-	6	NeuAcα-6Galβ-4Glcβ-NH-Gly-NH-DA	451	-	-
LNFP I-Gly-	7	Fucα-2Galβ-3GlcNAcβ-3Galβ-4Glc-NH-Gly-NH-DA	-	-	-
LSTb-Gly-	8	Galβ-3GlcNAcβ-3Galβ-4Glcβ-NH-Gly-NH-DA<br>| NeuAcα-6	4 766	-	-
DSLNT-Gly-	9	NeuAcα-3Galß-3GlcNAcß-3Galß-4Glcß-NH-Gly-NH-DA<br>| NeuAcα-6	4 597	-	-
DSMFLNH-Gly-	10	Galß-4GlcNAcß-6<br>| | Fucα-3 Galß-4Glcβ-NH-Gly-NH-DA<br>| NeuAcα-3Galß-3GlcNAcß-3<br>| NeuAcα-6	6 347	759	-
ChitoBiose-Gly-	11	GlcNAcβ-4GlcNAcβ-NH-Gly-NH-DA	-	-	-
Fuc(3)GlcNAc-Gly-	12	GlcNAcβ-NH-Gly-NH-DA<br>| Fucα-3	-	-	-
Fuc(6)GlcNAc-Gly-	13	Fucα-6GlcNAcβ-NH-Gly-NH-DA	-	-	-
DFuc(3,6)GlcNAc-Gly-	14	Fucα-6GlcNAcβ-NH-Gly-NH-DA<br>| Fucα-3	-	-	-

Man5-Gly-	15	Manα-6<br>| Manα-3Manα-6<br>| Manβ-4GlcNAcβ-4GlcNAcβ-NH-Gly-NH-DA<br>| Manα-3	-	-	-
Man6-Gly-	16	Manα-6<br>| Manα-3Manα-6<br>| Manβ-4GlcNAcβ-4GlcNAcβ-NH-Gly-NH-DA<br>| Manα-2Manα-3	-	-	-
NA2-Gly-	17	Galβ-4GlcNAcβ-2Manα-6<br>| Manβ-4GlcNAcβ-4GlcNAc-NH-Gly-NH-DA<br>| Galβ-4GlcNAcβ-2Manα-3	-	-	-
NA2F-Gly-	18	Galβ-4GlcNAcβ-2Manα-6 Fucα-6<br>| | Manβ-4GlcNAcβ-4GlcNAc-NH-Gly-NH-DA<br>| Galβ-4GlcNAcβ-2Manα-3	-	-	-
SM1a-C3-N-	19	Galβ-3GalNAcβ-4Galβ-4Glcβ-O-C3-NH-DA<br>| SU-3	-	-	-
SM1a(2S)-C3-N-	20	Galβ-3GalNAcβ-4Galβ-4Glcβ-O-C3-NH-DA<br>| SU-2	-	-	-
GalNAcα-ON-	21	GalNAcα-O-N=DA	-	-	-
GalNAcα-Ser-	22	GalNAcα-O-Ser-NH-DA	-	-	-
GalNAcα-Thr-	23	GalNAcα-O-Thr-NH-DA	-	-	-
GalNAcβ-Ser-	24	GalNAcβ-O-Ser-NH-DA	-	-	-
GalNAcβ-Thr-	25	GalNAcβ-O-Thr-NH-DA	-	-	-
GlcNAcβ-Ser-	26	GlcNAcβ-O-Ser-NH-DA	-	-	-
GlcNAcβ-Thr-	27	GlcNAcβ-O-Thr-NH-DA	-	-	-
STn-Ser-	28	NeuAcα-6GalNAcα-O-Ser-NH-DA	1 687	410	8 226
STn-Thr-	29	NeuAcα-6GalNAcα-O-Thr-NH-DA	543	-	789
Core 1-Ser-	30	Galβ-3GalNAcα-O-Ser-NH-DA	-	-	-

Core 1-Thr-	31	Galβ-3GalNAcα-O-Thr-NH-DA	-	-	-
SA1(2-3)-Core 1-Ser-	32	NeuAcα-3Galβ-3GalNAcα-O-Ser-NH-DA	-	-	-
SA1(2-3)-Core 1-Thr-	33	NeuAcα-3Galβ-3GalNAcα-O-Thr-NH-DA	852	-	-
SA1(2-6)-Core 1-Ser-	34	Galβ-3GalNAcα-O-Ser-NH-DA<br>NeuAcα-6	-	-	-
SA1(2-6)-Core 1-Thr-	35	Galβ-3GalNAcα-O-Thr-NH-DA<br>NeuAcα-6	224	-	-
SA2-Core 1-Ser-	36	NeuAcα-3Galβ-3GalNAcα-O-Ser-NH-DA<br>NeuAcα-6	748	-	-
SA2-Core 1-Thr-	37	NeuAcα-3Galβ-3GalNAcα-O-Thr-NH-DA<br>NeuAcα-6	436	-	-
Core 2-C3-N-	38	Galβ-3GalNAcα-O-C3-NH-DA<br>GlcNAcβ-6	-	-	-
Core 2-Thr-	39	Galβ-3GalNAcα-O-Thr-NH-DA<br>GlcNAcβ-6	40	-	-
Core 3-Ser-	40	GlcNAcβ-3GalNAcα-O-Ser-NH-DA	-	-	-
Core 3-Thr-	41	GlcNAcβ-3GalNAcα-O-Thr-NH-DA	1 450	549	-
Gal-Core 3-Ser-	42	Galβ-4GlcNAcβ-3GalNAcα-O-Ser-NH-DA	40	-	-
Gal(3S)-Core 3-Ser-	43	Gal(3S)β-4GlcNAcβ-3GalNAcα-O-Ser-NH-DA	151	16	-
GalNAc-Gal(3S)-Core 3-Ser-	44	GalNAcβ-4Gal(3S)β-4GlcNAcβ-3GalNAcα-O-Ser-NH-DA	-	-	-
GSC 967-Ser-	45	Galβ-3GalNAcβ-4Gal(3S)β-4GlcNAcβ-3GalNAcα-O-Ser-NH-DA	-	-	-
Core 4-C3-N-	46	GlcNAcβ-3GalNAcα-O-C3-NH-DA<br>GlcNAcβ-6	-	-	-
Core 4-Thr-	47	GlcNAcβ-3GalNAcα-O-Thr-NH-DA<br>GlcNAcβ-6	-	-	-
Man-PentaPep-	48	YAT(Man)AV-NH-DA[4]	-	-	-
Man-HexaPep-	49	CYAT(Man)AV-NMe-DA[4]	477	-	-
Man-UndecaPep-	50	Sp-TriEG-SQSLEET(Man)ISPR-NH-DA[3]	-	-	-

Hep-4-NAc-PhN-	51	GlcNAcα-4GlcAβ-4GlcNAcα-4GlcAβ1-O-Ph-NH-DA	1 106	-	-
Hep-4-NS-PhN-	52	GlcNSα-4GlcAβ-4GlcNSα-4GlcAβ1-O-Ph-NH-DA	904	-	-
Glc12-C5-N-	53	Glcβ-3Glcβ-3Glcβ-3Glcβ-[3Glcβ-3Glcβ]$_3$-3Glcβ-3Glcβ-O-C5-NH-DA	-	-	-
Glc15-C2-N-	54	Glcβ-3Glcβ-3Glcβ-3Glcβ-[3Glcβ-3Glcβ]$_3$-3Glcβ-3Glcβ-3Glcβ-3Glcβ-3Glcβ-O-C2-NH-DA	-	-	-
Glc13(B10)-	55	Glcβ-3Glcβ-3Glcβ-3Glcβ-[3Glcβ-3Glcβ]$_3$-3Glcβ-3Glcβ-O-C5-NH-DA<br>| Glcβ-6	-	-	-
Gal-C2-N-	56	Galβ-O-C2-NX-DA	-	-	-
Gal-Ph-N-	57	Galβ-O-Ph-NH-DA	-	-	-
Glc-C2-N-	58	Glcβ-O-C2-NX-DA	-	-	-
Man-C2-N-	59	Manβ-O-C2-NH-DA	-	-	-
Xyl-C2-N-	60	Xylβ-O-C2-NX-DA	-	-	-
**Cer, AO- and DH-NGLs**					
Lac-	61	Galβ-4Glc-AO	-	-	-
B-Tetra-T2-	62	Galα-3Galβ-4GlcNAc-AO<br>| Fucα-2	-	-	-
NeuAcα-(3')Lac-	63	NeuAcα-3Galβ-4Glc-AO	4 711	-	-
LNFP-I-	64	Fucα-2Galβ-3GlcNAcβ-3Galβ-4Glc-AO	-	-	-
LNFP-III-	65	Galβ-4GlcNAcβ-3Galβ-4Glc-DH<br>| Fucα-3	-	-	-
LSTb-	66	Galβ-3GlcNAcβ-3Galβ-4Glc-DH<br>| NeuAcα-6	5 610	-	-
H2 (with H2+Fuc)-	67	Fucα-2Galβ-4GlcNAcβ-3Galβ-4GlcNAcβ-3Galβ-4Glcβ-Cer*	-	-	-
TFiLNO-	68	Galβ-3GlcNAcβ-3Galβ-4GlcNAcβ-6<br>| | | Fucα-4 Fucα-3 Galβ-4Glc-DH<br>| Galβ-3GlcNAcβ-3<br>| Fucα-4	-	-	-
Asialo-GM1-Tetra-	69	Galβ-3GalNAcβ-4Galβ-4Glc-DH	-	-	-
SM1a-	70	Galβ-3GalNAcβ-4Galβ-4Glcβ-Cer<br>| SU-3	-	-	-

SB1a-	71	SU-3Galβ-3GalNAcβ-4Galβ-4Glcβ-Cer<br>| SU-3	-	-	-
GalNAc-Ser-	72	GalNAcα-Ser-DH	-	-	-
GalNAc-Thr-	73	GalNAcα-Thr-DH	-	-	-
BSM-Di-A1-	74	NeuGcα-6GalNAc-AO	633	-	-
BSM-Di-A2-	75	NeuAcα-6GalNAc-AO	3 112	-	-
Galβ-3GalNAc-	76	Galβ-3GalNAc-AO	-	-	-
Galβ-6GalNAc-	77	Galβ-6GalNAc-AO	-	-	-
DST-	78	NeuAcα-3Galβ-3GalNAc-AO<br>| NeuAcα-6	3 889	-	-
Curd-13-	79	Glcβ-3Glcβ-3Glcβ-3Glcβ-[3Glcβ-3Glcβ]$_3$-3Glcβ-3Glcβ-3Glc-AO*	-	-	-
Cello-3-	80	Glcβ-4Glcβ-4Glc-AO	-	-	-
GN2-	81	GlcNAcβ-4GlcNAc-AO	-	-	-
GN6-	82	GlcNAcβ-4GlcNAcβ-4GlcNAcβ-4GlcNAcβ-4GlcNAcβ-4GlcNAc-AO*	-	-	-

[1]The oligosaccharide probes are all lipid-linked, neoglycolipids (NGLs) or glycosylceramides and are sorted by glycan backbone type referred to a position as in the binding charts in **Figure 4**.

[2]Abbreviations are as follows: DA, NGLs prepared using a new aldehyde-terminating lipid reagent to conjugate with amino-terminating glycans; NX, NH or NMe; C2, -CH_2-CH_2-; C3, -CH_2-CH_2-CH_2-; C5, -CH_2-CH_2-CH_2-CH_2-CH_2-; Gly, glycine; Ser, serine; Thr, threonine; Ph, phenyl; Gly, glycine. For the control probes: DH, NGLs prepared from reducing oligosaccharides by reductive amination with the amino lipid, 1,2-dihexadecyl-sn-glycero-3-phosphoethanolamine (DHPE)(Chai et al. 2003); AO, NGLs prepared from reducing oligosaccharides by oxime ligation with an aminooxy (AO) functionalized DHPE(Liu et al. 2007); Cer, natural glycolipids with various ceramide moieties.

[3]Means of fluorescence intensities of duplicate spots printed at the high level of probe arrayed (5fmol/spot); –, less than 1.

[4]*O*-mannosyl glycopeptide.

*Asterisks indicate that major components are depicted.

References

Bartels MF, Winterhalter PR, Yu J, et al. 2016. Protein O-mannosylation in the murine brain: Occurrence of Mono-O-Mannosyl glycans and identification of new substrates. *PLoS One*. 11: e0166119.

Chai W, Hounsel EF, Cashmore GC, Rosankiewicz JR, Feeney J, Lawson AM. 1992. Characterisation by mass spectrometry and 1H-NMR of novel hexasaccharides among the acidic O-linked carbohydrate chains of bovine submaxillary mucin. *Eur J Biochem*.207: 973–80.

Chai W, Stoll MS, Galustian C, et al. 2003. Neoglycolipid technology: Deciphering information content of glycome. *Methods Enzymol*. 362: 160–195.

Gao C, Liu Y, Zhang H, et al. 2014. Carbohydrate sequence of the prostate cancer-associated antigen F77 assigned by a mucin O-glycome designer array. *J Biol Chem.* 289: 16462–16477.

Likhosherstov L.M., Novikova O.S., Yamskov I.A. PVE. 2012. Synthesis of N-glycyl-beta-glycopyranosylamines, human milk fucooligosaccharide derivatives. *Russ Chem Bull*. 61: 1816–1817.

Likhosherstov LM, Novikova OS, Malysheva NN, et al. 2015. Synthesis of mono- and di-α-l-fucosylated 2-acetamido-2-deoxy-N-glycyl-β-d-glucopyranosylamines modeling N-glycoprotein carbohydrate-peptide bond region based on 2-acetamido-N-(N-tert-butyloxycarbonylglycyl)-2-deoxy-β-d-glucopyranosylamine. *Russ Chem Bull*. 64: 1445–1450.

Likhosherstov LM, Novikova OS, Sakharov AM, et al. 2016. Synthesis of N-aminoacyl-β-glycopyranosylamines — derivatives of natural sialooligosaccharides. *Russ Chem Bull*. 65: 1617–1624.

Liu Y, Feizi T, Campanero-Rhodes MA, et al. 2007. Neoglycolipid Probes Prepared via Oxime Ligation for Microarray Analysis of Oligosaccharide-Protein Interactions. *Chem Biol*. 14: 847–859.

Liu Y, Childs RA, Palma AS, et al. 2012. Neoglycolipid-Based Oligosaccharide Microarray System: Preparation of NGLs and Their Noncovalent Immobilization on Nitrocellulose-Coated Glass Slides for Microarray Analyses BT - Carbohydrate Microarrays: Methods and Protocols. In: Chevolot Y (ed). Totowa, NJ: Humana Press, pp. 117–136.

Nishimura S-I, Kuzuhara H, Takiguchi Y, et al. 1989. Peracetylated chitobiose: Preparation by specific degradations of chitin, and chemical manipulations. *Carbohydr Res*. 194: 223–231.

Palma AS, Liu Y, Childs RA, et al. 2011. The human epithelial carcinoma antigen recognized by monoclonal antibody AE3 is expressed on a sulfoglycolipid in addition to neoplastic mucins. *Biochem Biophys Res Commun*. 408: 548–552.

Palma AS, Liu Y, Zhang H, et al. 2015. Unravelling glucan recognition systems by glucome microarrays using the designer approach and mass spectrometry. *Mol Cell Proteomics*. 14: 974–988.

Parkkinen J, Finne J. 1983. Isolation and structural characterization of five major sialyloligosaccharides and a sialylglycopeptide from normal human urine. *Eur J Biochem*. 136: 355–361.

Piskarev VE, Shuster AM, Gabibov AG, et al. 1990. A novel preparative method for the isolation of avidin and riboflavin-binding glycoprotein from chicken egg-white by the use of high-performance liquid chromatography. *Biochem J*. 265: 301–304.

Vendele I, Willment JA, Silva LM, et al. 2020. Mannan detecting C-type lectin receptor probes recognise immune epitopes with diverse chemical, spatial and phylogenetic heterogeneity in fungal cell walls. *PLoS Pathog*. 16: 1–29.

Weishaupt MW, Matthies S, Seeberger PH. 2013. Automated solid-phase synthesis of a β-(1,3)-glucan dodecasaccharide. *Chem - A Eur J*. 19: 12497–12503.

Weishaupt MW, Hahm HS, Geissner A, et al. 2017. Automated glycan assembly of branched β-(1,3)-glucans to identify antibody epitopes. *Chem Commun.* 53: 3591–3594.
